# First myocardial infarction: risk factors, symptoms, and medical therapy

**DOI:** 10.1093/eurheartj/ehaf390

**Published:** 2025-07-03

**Authors:** Nick S Nurmohamed, Quyen Ngo-Metzger, Pam R Taub, Kausik K Ray, Gemma A Figtree, Marc P Bonaca, Judith A Hsia, Santosh Angadageri, James P Earls, Fatima Rodriguez, Alexander T Sandhu, James K Min, Udo Hoffmann, David J Maron, Deepak L Bhatt

**Affiliations:** Department of Cardiology, Amsterdam UMC, Vrije Universiteit Amsterdam, Amsterdam, the Netherlands; Department of Vascular Medicine, Amsterdam UMC, University of Amsterdam, Amsterdam, the Netherlands; Division of Cardiology, The George Washington University School of Medicine, Washington, DC, USA; Department of Health Systems Science, Kaiser Permanente Bernard J. Tyson School of Medicine, Pasadena, CA, USA; Division of Cardiovascular Diseases, Department of Medicine, University of California, San Diego, CA, USA; Imperial Centre for Cardiovascular Disease Prevention, School of Public Health, Imperial College London, London, United Kingdom; Faculty of Medicine & Health, University of Sydney, Camperdown, NSW, Australia; Department of Medicine, Cardiology and Vascular Medicine, University of Colorado School of Medicine, Aurora, CO, USA; Department of Medicine, Cardiology and Vascular Medicine, University of Colorado School of Medicine, Aurora, CO, USA; Data Analytics & Insights, Clarivate Analytics US LLC, Toronto, ON, Canada; Cleerly Inc., Denver, CO, USA; Department of Medicine, Stanford University School of Medicine, Stanford, CA, USA; Department of Medicine, Stanford University School of Medicine, Stanford, CA, USA; Cleerly Inc., Denver, CO, USA; Cleerly Inc., Denver, CO, USA; Department of Medicine, Stanford University School of Medicine, Stanford, CA, USA; Mount Sinai Fuster Heart Hospital, Icahn School of Medicine at Mount Sinai, 1 Gustave Levy Place, Box 1030, New York, NY 10029, USA

**Keywords:** Acute coronary syndromes, Standard modifiable risk factors, Myocardial infarction, Prevention, Real-world data

## Abstract

**Background and Aims:**

Despite the implementation of clinical risk algorithms based on traditional risk factors, the global burden of atherosclerotic cardiovascular disease has continued to rise over the past decades. There are few real-world data on prevalence of both symptoms and risk factors prior to myocardial infarction (MI). This study aimed to investigate the prevalence of documented coronary artery disease risk factors, documented symptoms, physician visits, and preventive therapy uptake prior to first MI.

**Methods:**

In this retrospective cohort study, US patients ≥18 years with a first MI [International Classification of Diseases, 10th Revision (ICD-10) definition] between 1 January 2017 and 30 September 2022 were included from the Clarivate Real-World Data Product that links electronic health records, medical claims, and pharmacy claims from 98% of government and commercial health insurance plans in the US. Prevalence of previously ICD-10 documented cardiac symptoms and standard modifiable risk factors (SMuRFs), physician visits, and use of preventive medical therapy were assessed prior to MI.

**Results:**

The study identified 4 657 412 patients with a first MI (2017–2022), with a median age of 70 years; 42.3% were women. Prior to MI, 50.5% of patients had no documented symptoms, 18.0% had no SMuRFs, 22.2% did not have documented physician visits, and 63.4% were not prescribed any preventive therapy. Individuals ≤60 years and men were less likely to have documented symptoms and SMuRFs, had lower frequency of primary care physician visits, used less preventive therapy, and had more frequent occurrence of ST-elevation MI than individuals >60 years and women, respectively.

**Conclusions:**

In a large real-world dataset, half of the patients with first MI did not have documented antecedent symptoms, and approximately 1 in 5 had no previously identified SMuRFs. The majority of those who visited a physician prior to the MI with identified SMuRFs and symptoms were not using any preventive medical therapy. These findings highlight an urgent unmet need for improved tools to identify patients at risk of MI who may benefit from preventive therapy.

## Structured Graphical Abstract

**Structured Graphical Abstract:**
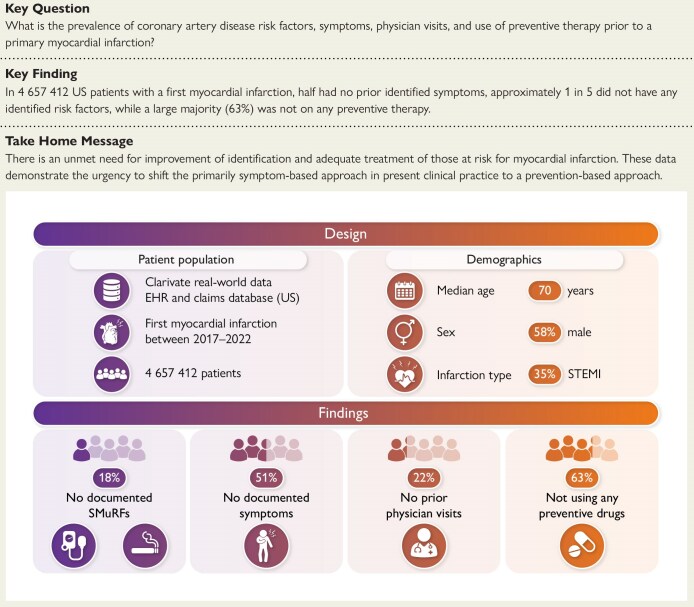
Identification and treatment of patients prior to first myocardial infarction. EHR, electronic health records; MI, myocardial infarction; SMuRFs, standard modifiable risk factors; STEMI, ST-elevation myocardial infarction; US, United States.

**See the editorial comment for this article ‘Failing to prevent heart attacks: what will it take to stem the tide?’, by S.J. Nicholls, https://doi.org/10.1093/eurheartj/ehaf414.**

## Introduction

Annually, more than 20 million people worldwide die from cardiovascular disease, representing an increase of 60% over 30 years, of which the majority is caused by atherosclerotic cardiovascular disease (ASCVD).^1^ Given the rise in behavioural risk factors such as obesity and physical inactivity, without intervention, the number of cardiovascular deaths is expected to continue increasing in the next decades.^1^

The current population-based strategy for primary prevention of ASCVD uses clinical risk algorithms to estimate ASCVD risk followed by risk factor modification.^2,3^ These clinical risk scores—including the Pooled Cohort Equations, Systematic Coronary Risk Evaluation 2 system, and the Framingham risk score^2,4,5^—are based on traditional cardiovascular risk factors such as age, sex, smoking, hypertension, and plasma cholesterol levels. Adding to observations from cohort studies showing limited discriminatory value and accuracy of these algorithms for predicting future events, the real-world use and benefit of these algorithms are further questioned by the observed rise in ASCVD incidence in recent years.

Adding to a foundation of statin and ezetimibe therapy, novel medications such as monoclonal antibody proprotein convertase subtilisin/kexin type 9 inhibitors and inclisiran,^6^ icosapent ethyl,^7^ sodium-glucose cotransporter 2 inhibitors,^8^ glucagon-like peptide-1 receptor agonists,^9^ bempedoic acid,^10^ and low-dose rivaroxaban^11^ have provided physicians ample tools to treat patients across the full spectrum of risk and reduce residual risk. In an era of this broad pharmacological armamentarium of risk-lowering therapies, effective and timely identification of patients at the highest ASCVD risk events poses one of the key challenges in current ASCVD prevention.

To date, there are few real-world data on prevalence of identified risk factors and symptoms prior to myocardial infarction (MI). Whether conventional clinical risk scoring followed by risk factor modification, as is the current standard of care, effectively identifies at-risk individuals prior to first MI has not been sufficiently explored in real-world practice. It is unknown whether individuals with risk factors or symptoms undergo timely ASCVD risk stratification and whether they receive adequate therapy to prevent the occurrence of ASCVD events such as MI. The aim of the current study was to assess the prevalence of symptoms, risk factors, physician visits, and preventive medication use in a large, combined database consisting of electronic health records (EHR) and medical claims from 5 million US patients with a first MI.

## Methods

### Study design, data sources, and population

In this real-world retrospective cohort study, data were obtained from the Clarivate Real-World Data Product (Clarivate RWD) that links EHR, medical claims, and pharmacy claims covering approximately 98% of government and commercial health insurance plans in the United States and covers >300 million lives in total.^12–14^ EHR data were sourced directly from providers and linked to open claims data from transactional clearing houses by a Health Insurance Portability and Accountability Act-compliant encrypted patient key generated by a third party. The assigned patient key allowed for longitudinal tracking of patients across time and all data sources, providing information on patient demographic, geographic location, diagnoses and comorbidities, clinical tests, and therapeutic interventions.

We included patients ≥18 years who suffered a first MI according to International Classification of Diseases, 10th Revision (ICD-10) codes (eTable 1) between 1 January 2017 and 30 September 2022. A total of 5 068 740 patients with at least one MI claim were identified (see Supplementary data online, *Figure S1*). Patients with a prior MI, defined as a previous ICD-10 coded MI starting from 1 January 2016, or a ICD-9 coded MI (see Supplementary data online, *Table S1*) between 1 January 2014 and 31 December 2015, were excluded from the analysis.

### Definition of exposures and outcomes: myocardial infarction, risk factors, symptoms, and medical therapy

Myocardial infarctions were categorized as either a ST-elevation MI (STEMI) or non-ST-elevation MI according to the ICD-10 definition (see Supplementary data online, *Table S1*). Previous validation studies have shown high accuracy of ICD-10 for identifying patients with MI.^15,16^

The prevalence of standard modifiable coronary artery disease risk factors, symptoms, physician visits, and preventive medical therapy were assessed at 6 months prior to MI. Standard modifiable risk factors (SMuRFs) were defined as dyslipidaemia, hypertension, smoking, family history of MI, diabetes (type 1, 2, or gestational), obesity, and alcohol abuse according to ICD-10 codes. The ICD-10 codes used are shown in Supplementary data online, *Table S2*. The ICD-10 coded list of possible cardiac symptoms included chest, arm, back, shoulder or jaw pain, arrhythmias, dyspnoea, dyspepsia, fatigue, and dizziness, is shown in Supplementary data online, *Table S3*. Prior analyses have demonstrated the utility of ICD-10 codes from claims databases for symptom identification.^17,18^ Visits to primary care physicians (PCP) and cardiologists were determined according to medical claims of visits to a PCP and cardiologists, respectively. The use of preventive medical therapy was determined using the pharmacy claims; only paid claims during the study period were included in the analysis. The preventive medical therapy definition included drugs for lipid lowering, blood pressure lowering, glycaemic control, and antithrombotic therapy (see Supplementary data online, *Table S4*).

### Statistical analysis

Data are presented as median (Q1–Q3). Categorical variables are expressed as absolute numbers and percentages. Independent sample *t*-tests and chi-squared tests were used where appropriate. Descriptive data were stratified according to age and sex. When stratified by sex, patients with unknown sex (0.04%) were excluded from the analysis. Unadjusted and adjusted logistic regression models were used to assess the relationship between demographics (age, sex, and geographic origin; independent variables) and presence of documented symptoms, SMuRFs, physician visits, use of preventive medical therapy and STEMI (dependent variable). In case of unknown sex, patients were considered male in the logistic regression models. All statistical analyses were performed using Python (version 3.10) and RStudio (version 4.3.2).

## Results

### Patient population

The study identified 4 657 412 patients with a first MI after exclusion of 411 328 patients with prior MI (see Supplementary data online, *Figure S1*; *Table 1*). Included patients had a median age of 70 (60–80) years, and 1 968 081 (42.3%) were women. The majority of patients were from an urban environment [3 859 869 (82.9%)]. A total of 164 611 (3.5%) patients underwent prior revascularization. In the majority of patients (3 020 340; 64.9%), MI was non-ST elevation, while 1 637 072 (35.1%) patients experienced an STEMI.

**Table 1:** Patient characteristics

Characteristic	Overall (*n* = 4 657 412)
Age at MI, years	70 (60–80)
Sex	
Male	2 687 600 (57.7%)
Female	1 968 081 (42.3%)
Unknown	1731 (0.0%)
Documented SMuRFs	
One or more SMuRFs	3 817 522 (82.0%)
Dyslipidaemia	2 969 073 (63.7%)
Type 2 diabetes	1 934 636 (41.5%)
Obesity	1 344 724 (28.9%)
Hypertension	1 241 403 (26.7%)
Nicotine dependence	899 259 (19.3%)
Family history of CAD	390 259 (8.4%)
Alcohol abuse	254 199 (5.5%)
Type 1 diabetes	230 169 (4.9%)
Gestational diabetes	3444 (0.1%)
Documented symptoms	
Yes	2 304 514 (49.5%)
No	2 352 898 (50.5%)
Use of preventive medical therapy	
One or more drugs	1 702 364 (36.6%)
Statins	1 008 909 (21.7%)
Beta blockers	923 696 (19.8%)
Diuretics	730 090 (15.7%)
ACE inhibitors	698 444 (15.0%)
Calcium channel blockers	624 937 (13.4%)
Antihyperglycaemics	624 159 (13.4%)
Antiplatelet drugs	448 391 (9.6%)
Angiotensin II receptor blockers	438 341 (9.4%)
Oral anticoagulants	291 006 (6.2%)
Nitrates	283 931 (6.1%)
Vasodilators	119 362 (2.6%)
Ezetimibe	55 504 (1.2%)
Other lipid-lowering drugs	26 796 (0.6%)
PCSK9 inhibitors	10 403 (0.2%)
Visit to physician prior to MI	
Yes	3 622 711 (77.8%)
No	1 034 701 (22.2%)
Visit to PCP before MI	
Yes	3 550 594 (76.2%)
No	1 106 818 (23.8%)
Visit to cardiologist before MI	
Yes	1 750 785 (37.6%)
No	2 906 627 (62.4%)
Type of MI	
STEMI	1 637 072 (35.1%)
NSTEMI	3 020 340 (64.9%)
Previous revascularization	
Any	164 611 (3.5%)
PCI	130 462 (2.8%)
CABG	32 397 (0.7%)
Unspecified	14 760 (0.3%)
Geographic area	
Urban	3 859 869 (82.9%)
Rural	770 626 (16.5%)
Other	26 917 (0.5%)

Median (Q1–Q3). ACE, angiotensin-converting enzyme; CABG, coronary artery bypass grafting; CAD, coronary artery disease; PCI, percutaneous coronary intervention; PCP, primary care physician; PCSK9, proprotein convertase subtilisin-kexin type 9; MI, myocardial infarction; NSTEMI, non-ST-elevation myocardial infarction; SMuRF, standard modifiable risk factor; STEMI, ST-elevation myocardial infarction.

### Prevalence of documented symptoms and risk factors, medication use, and physician visits

The majority of patients (3 817 522; 82.0%) had at least one or more documented SMuRFs (*Table 1*). Half of the included patients had no documented symptoms prior to their MI [2 352 898 (50.5%)]. The most frequently observed SMuRFs were dyslipidemia (2 969 073; 63.7%), type 2 diabetes (1 934 636; 41.5%), obesity (1 344 724; 28.9%), and hypertension (1 241 403; 26.7%). Preventive medical therapy was used in 1 702 364 (36.6%) patients; mostly statins (1 008 909; 21.7%), beta blockers (923 696; 19.8%), diuretics (730 090; 15.7%), ACE inhibitors (698 444; 15.0%), and calcium channel blockers (624 937; 13.4%), while 624 159 (13.4%) patients used glucose-lowering medication. Before occurrence of the MI, a total of 3 622 711 (77.8%) patients had visited their physician: 3 550 594 (76.2%) patients had visited their PCP, while a minority had a documented visit to a cardiologist (1 750 785; 37.6%).

**Figure 1:**
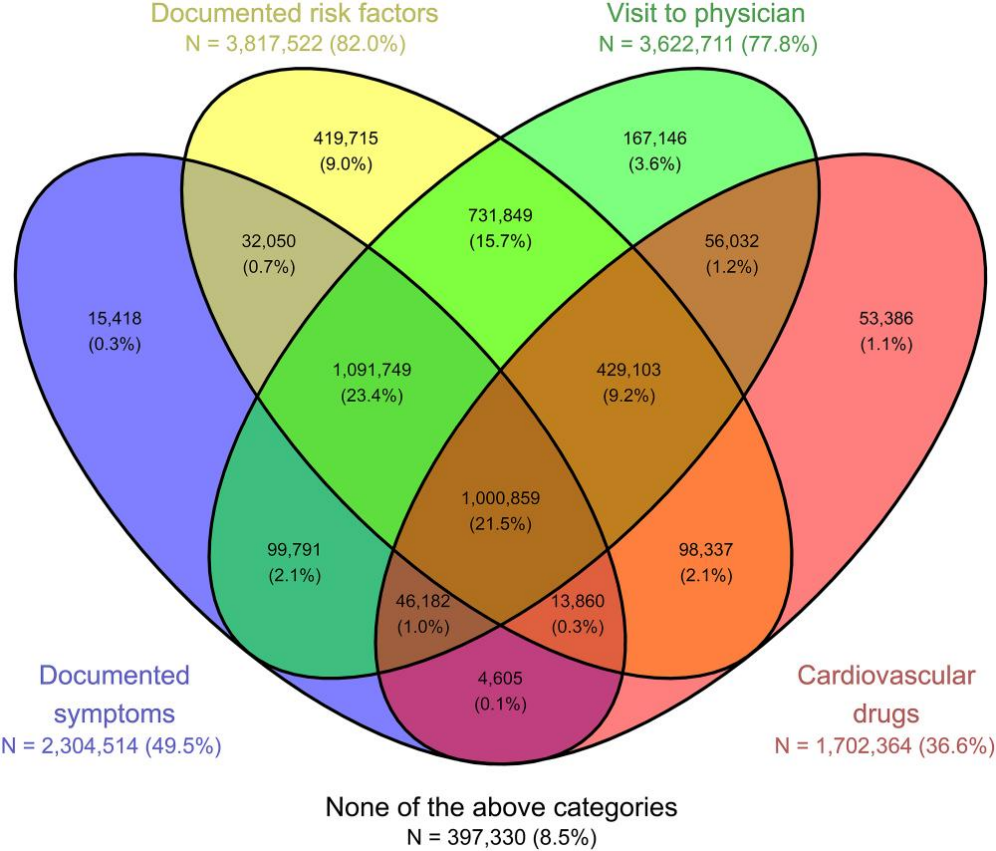
Association between documented risk factors, documented symptoms, physician visits, and use of cardiovascular drugs. Venn diagram of documented risk factors, symptoms, physician visits, and use of cardiovascular drugs prior to myocardial infarction

Of the patients with documented SMuRFs, 2 138 518 (56.0%) also had documented symptoms (*Figure 1*). A majority of the patients who visited a physician prior to their MI and had documented SMuRFs present did not use any cardiovascular drugs [1 823 598 (56.0%); *Figure 1*]. Moreover, of those who visited a physician (PCP and/or cardiologist) with documented symptoms, a majority did not use any cardiovascular drugs [1 191 540 (53.2%); *Figure 1*]. Of the patients with both documented symptoms and SMuRFs and a physician visit prior to their MI, 1 091 749 (52.2%) patients did not use any cardiovascular drugs (*Figure 1*).

### Prevalence of risk factors, symptoms, physician visits, and myocardial infarction type in older vs younger patients

A total of 1 187 306 (25.5%) patients were aged 60 years or younger, with a median age of 53 (47–57) years (*Table 2*). Patients older than 60 years (3 470 106; 74.5%) had a median age of 75 (68–83) years. Individuals aged older than 60 years were more likely to have documented symptoms: 1 812 039 (52.2%) vs 492 475 (41.5%) in patients younger than 60 years [adjusted OR 1.196 (95% CI 1.194–1.197) per 10-year increase in age; *P* < .001; *Table 3*]. Compared with younger patients, patients older than 60 years were more likely to have documented SMuRFs [2 916 608 (84.1%) vs 900 914 (75.9%); adjusted OR 1.208 (95% CI 1.206–1.210) per 10-year increase in age]. Furthermore, older patients were more likely to have visited a physician prior to their MI [2 822 257 (81.3%) vs 800 454 (67.4%); adjusted OR 1.341 (95% CI 1.339–1.344) per 10-year increase in age; *P* < .001]. Older patients were more likely to have used preventive medical therapy: 1 343 956 (38.7%) did use preventive therapy prior to the MI vs 358 408 (30.2%) younger patients [adjusted OR 1.151 (1.149–1.153) per 10-year increase in age; *P* < .001]. Finally, older patients were less likely to experience a STEMI [1 132 637 (32.6%)] than the younger patients, of whom 504 435 (42.5%) had a STEMI [adjusted OR 0.836 (95% CI 0.835–0.838) per 10-year increase in age; *P* < .001].

**Table 2:** Differences in patient characteristics, symptoms, risk factors, preventive therapy, and myocardial infarction type according to age and sex

Characteristic	Age		Sex	
	≤60 years *n* = 1 187 306	>60 years *n* = 3 470 106	Female *n* = 1 968 081	Male *n* = 2 687 600
Age at MI, years	53 (47–57)	75 (68–83)	73 (62–83)	68 (59–78)
Sex				
Male	768 515 (64.7%)	1 919 085 (55.3%)		
Female	418 298 (35.2%)	1 549 783 (44.7%)		
Unknown	493 (0.0%)	1238 (0.0%)		
Documented symptoms				
Yes	492 475 (41.5%)	1 812 039 (52.2%)	1 096 604 (55.7%)	1 207 873 (44.9%)
No	694 831 (58.5%)	1 658 067 (47.8%)	871 477 (44.3%)	1 479 727 (55.1%)
Documented SMuRFs				
One or more SMuRFs	900 914 (75.9%)	2 916 608 (84.1%)	1 632 486 (82.9%)	2 184 552 (81.3%)
Dyslipidaemia	578 682 (48.7%)	2 390 391 (68.9%)	1 274 236 (64.7%)	1 694 600 (63.1%)
Type 2 diabetes	393 136 (33.1%)	1 541 500 (44.4%)	835 409 (42.4%)	1 098 994 (40.9%)
Obesity	387 112 (32.6%)	957 612 (27.6%)	622 084 (31.6%)	722 572 (26.9%)
Hypertension	189 526 (16.0%)	1 051 877 (30.3%)	559 711 (28.4%)	681 590 (25.4%)
Nicotine dependence	365 015 (30.7%)	534 244 (15.4%)	347 766 (17.7%)	551 440 (20.5%)
Family history of CAD	110 256 (9.3%)	279 803 (8.1%)	173 295 (8.8%)	216 748 (8.1%)
Alcohol abuse	103 705 (8.7%)	150 494 (4.3%)	63 432 (3.2%)	190 752 (7.1%)
Type 1 diabetes	63 346 (5.3%)	166 823 (4.8%)	108 137 (5.5%)	122 023 (4.5%)
Gestational diabetes	3310 (0.3%)	134 (0.0%)		
Use of preventive medical therapy				
One or more drugs	358 408 (30.2%)	1 343 956 (38.7%)	784 016 (39.8%)	918 347 (34.2%)
Statins	189 821 (16%)	819 088 (23.6%)	450 847 (22.9%)	558 061 (20.8%)
Beta blockers	173 396 (14.6%)	750 300 (21.6%)	429 646 (21.8%)	494 049 (18.4%)
ACE inhibitors	163 734 (13.8%)	534 710 (15.4%)	302 336 (15.4%)	396 108 (14.7%)
Antihyperglycaemics	138 833 (11.7%)	485 326 (14.0%)	282 518 (14.4%)	341 641 (12.7%)
Diuretics	130 195 (11%)	599 895 (17.3%)	374 228 (19%)	355 862 (13.2%)
Calcium channel blockers	115 103 (9.7%)	509 834 (14.7%)	308 502 (15.7%)	316 434 (11.8%)
Antiplatelet drugs	93 519 (7.9%)	354 872 (10.2%)	188 854 (9.6%)	259 537 (9.7%)
Angiotensin II receptor blockers	74 162 (6.2%)	364 179 (10.5%)	223 150 (11.3%)	215 191 (8.0%)
Nitrates	48 209 (4.1%)	235 722 (6.8%)	120 256 (6.1%)	163 675 (6.1%)
Oral anticoagulants	37 587 (3.2%)	253 419 (7.3%)	131 570 (6.7%)	159 436 (5.9%)
Vasodilators	24 727 (2.1%)	94 635 (2.7%)	58 753 (3%)	60 609 (2.3%)
Ezetimibe	8114 (0.7%)	47 390 (1.4%)	26 226 (1.3%)	29 278 (1.1%)
Other lipid-lowering drugs	4437 (0.4%)	22 359 (0.6%)	13 951 (0.7%)	12 845 (0.5%)
PCSK9 inhibitors	1454 (0.1%)	8949 (0.3%)	4788 (0.2%)	5615 (0.2%)
Visit to physician prior to MI				
Yes	800 454 (67.4%)	2 822 257 (81.3%)	1 624 562 (82.5%)	1 998 047 (74.3%)
No	386 852 (32.6%)	647 849 (18.7%)	343 519 (17.5%)	689 553 (25.7%)
Visit to PCP before MI				
Yes	781 389 (65.8%)	2 769 205 (79.8%)	1 600 817 (81.3%)	1 949 693 (72.5%)
No	405 917 (34.2%)	700 901 (20.2%)	367 264 (18.7%)	737 907 (27.5%)
Visit to cardiologist before MI				
Yes	322 512 (27.2%)	1 428 273 (41.2%)	784 305 (39.9%)	966 444 (36%)
No	864 794 (72.8%)	2 041 833 (58.8%)	1 183 776 (60.1%)	1 721 156 (64%)
Type of MI				
STEMI	504 435 (42.5%)	1 132 637 (32.6%)	617 493 (31.4%)	1 019 000 (37.9%)
NSTEMI	682 871 (57.5%)	2 337 469 (67.4%)	1 350 588 (68.6%)	1 668 600 (62.1%)
Previous revascularization				
Any	31 158 (2.6%)	133 453 (3.8%)	53 845 (2.7%)	110 763 (4.1%)
PCI	24 729 (2.1%)	105 733 (3.0%)	44 563 (2.3%)	85 897 (3.2%)
CABG	6599 (0.6%)	25 798 (0.7%)	9172 (0.5%)	23 225 (0.9%)
Unspecified	2228 (0.2%)	12 532 (0.4%)	3387 (0.2%)	11 372 (0.4%)
Geographic area				
Urban	992 973 (83.6%)	2 866 896 (82.6%)	1 635 871 (83.1%)	2 222 555 (82.7%)
Rural	189 518 (16.0%)	581 108 (16.7%)	320 842 (16.3%)	449 527 (16.7%)
Other	4815 (0.4%)	22 102 (0.6%)	11 368 (0.6%)	15 518 (0.6%)

Patients with unknown sex were not included in the columns stratified by sex. Median (Q1–Q3). Mean ± SD; *n* (%). ACE, angiotensin-converting enzyme; CABG, coronary artery bypass grafting; CAD, coronary artery disease; MI, myocardial infarction; NSTEMI, non-ST-elevation myocardial infarction; PCI, percutaneous coronary intervention; PCP, primary care physician; PCSK9, proprotein convertase subtilisin-kexin type 9; SMuRF, standard modifiable risk factor; STEMI, ST-elevation myocardial infarction.

**Table 3:** Association between age, sex, geographic area and symptoms, risk factors, preventive therapy, and myocardial infarction type

	Documented symptoms		Documented SMuRFs		Physician visit		Use of cardiovascular drugs		STEMI	
	OR (95% CI)	*P*-value	OR (95% CI)	*P*-value	OR (95% CI)	*P*-value	OR (95% CI)	*P*-value	OR (95% CI)	*P*-value
Age, per 10 years										
Univariable	1.218 (1.216–1.220)	<.001	1.215 (1.213–1.217)	<.001	1.364 (1.362–1.366)	<.001	1.164 (1.162–1.165)	<.001	0.828 (0.827–0.829)	<.001
Multivariable	1.196 (1.194–1.197)	<.001	1.208 (1.206–1.210)	<.001	1.341 (1.339–1.344)	<.001	1.151 (1.149–1.153)	<.001	0.836 (0.835–0.838)	<.001
*Male sex*										
Univariable	0.648 (0.646–0.650)	<.001	0.891 (0.886–0.895)	<.001	0.611 (0.608–0.614)	<.001	0.783 (0.780–0.786)	<.001	1.336 (1.330–1.341)	<.001
Multivariable	0.668 (0.665–0.670)	<.001	0.933 (0.929–0.938)	<.001	0.654 (0.651–0.657)	<.001	0.809 (0.806–0.812)	<.001	1.266 (1.261–1.271)	<.001
*From urban area*										
Univariable	1.018 (1.013–1.023)	<.001	1.021 (1.014–1.027)	<.001	1.057 (1.051–1.063)	<.001	0.725 (0.721–0.728)	<.001	1.098 (1.093–1.104)	<.001
Multivariable	1.025 (1.020–1.030)	<.001	1.032 (1.025–1.038)	<.001	1.073 (1.066–1.079)	<.001	0.724 (0.720–0.728)	<.001	1.096 (1.090–1.101)	<.001

Logistic regression models were adjusted for age, sex, and geographic origin. SMuRF, standard modifiable risk factor; STEMI, ST-elevation myocardial infarction.

### Prevalence of risk factors, symptoms, physician visits, and myocardial infarction type in men compared with women

Men comprised 57.7% of the study population and were slightly younger than women: 68 (59–78) vs 73 (62–83) years (mean age 67.4 vs 70.7 years), respectively (*Table 2*). Men were less likely to have documented symptoms than women [1 207 873 (44.9%) vs 1 096 604 (55.7%); adjusted OR 0.668 (95% CI 0.665–0.670); *P* < .001; *Table 3*]. Men were less likely to have visited a physician prior to the MI than women [1 998 047 (74.3%) vs 1 624 562 (82.5%); adjusted OR 0.654 (95% CI 0.651–0.657); *P* < .001]. The number of men with documented SMuRFs [2 184 552 (81.3%)] was also lower than the number of women [1 632 486 (82.9%)] with documented SMuRFs [adjusted OR 0.933 (95% CI 0.929–0.938); *P* < .001], and men were less likely to use preventive medical therapy prior to MI [918 347 (34.2%)] compared with women [784 016 (39.8%); adjusted OR 0.809 (95% CI 0.806–0.812); *P* < .001]. With respect to type of MI, 1 019 000 (37.9%) men experienced a STEMI, which was more frequent compared with the 617 493 (31.4%) women who had a documented STEMI [adjusted OR 1.266 (95% CI 1.261–1.271); *P* < .001].

## Discussion

Leveraging data from 4 657 412 patients, we show that half of patients did not experience any antecedent symptoms prior to a first MI. Furthermore, an important proportion of patients did not visit any physician while the majority were not prescribed any preventive therapy prior to the event. The number of patients not visiting a physician or receiving preventive therapy was higher in asymptomatic and younger patients. Collectively, these data illustrate the urgent unmet need for improved tools to identify patients at risk of MI who may benefit from preventive therapy (*Structured Graphical Abstract*).

To our knowledge, this is the largest study reporting on the prevalence of cardiac symptoms prior to MI to date. We observed that especially young patients more often lacked identified SMuRFs and that a significant part of these patients did not have a documented visit to any physician prior to the MI occurrence during the study period. The notion that half of patients did not have any documented symptoms in conjunction with the frequent absence of physician visits prior to their MI—thus absence of a potential opportunity for cardiovascular risk assessment and management—underscores the need to shift the medical practice paradigm more effectively from a symptom-based approach to a prevention-based approach. The current approach also disproportionally impacts younger individuals at risk for MI, as our data showed that they have less SMuRFs, are more likely to lack symptoms and thus are relatively undertreated. In order to effectively reduce the cardiovascular disease burden in the young and asymptomatic patients not presenting at the outpatient clinic prior to an event, a different preventive screening approach may be needed.

Risk factors underpinning current clinical algorithms, including standard modifiable components of smoking, hypertension, diabetes, and hypercholesterolaemia (SMuRFs) have long been identified in large population studies to associate with incidence of ASCVD; therefore, guidelines have recommended routine risk stratification.^2,3,19^ Despite the fact that 4 out of 5 MI patients had at least one risk factor, the burden of cardiovascular events as illustrated by more than 5 million MIs observed in the study period of almost 6 years remains high, in line with the previously reported annual US incidence of ∼805 000 MIs.^20^ This is not surprising, given less than half of the SMuRF-positive patients with MI were treated with preventive medication in the current study, aligned with prior data on statin prescription prior to MI.^21^ Even in patients with both documented SMuRFs and cardiac symptoms at a physician visit, the majority did not receive preventive medical therapy. Additionally, the 18% of the patients experiencing MI lacking any documented SMuRFs—a proportion similar to reports from previous large registries and studies investigating the prevalence of cardiovascular risk factors^22–27^—would not be identified by a risk factor-based approach at all. Combined, these findings demonstrate the limitations of a preventive approach based on risk factors, which are associated with cardiovascular events on a population level but lack discriminatory power to identify individuals at the highest risk for imminent ASCVD.^28,29^ The inability to accurately detect high-risk atherosclerosis in current clinical practice is highlighted further by the many patients who experience out-of-hospital sudden cardiac death as their first and often final cardiovascular event, who were not included in the current study.^30^

We observed significant differences between women and men. Women were more likely to visit a PCP before the event, received more preventive therapy, and were less likely to experience STEMI compared with men. Interestingly, the proportion of women in the current analysis restricted to patients with MI was 42%, significantly higher than previous reports describing a proportion of women between 29% and 32% among patients with acute MI.^31–33^ Additionally, we found that women were approximately 3 years older when experiencing a first MI and were more likely to experience symptoms prior to MI than men, which has been shown previously in smaller studies,^32,34,35^ although we did not investigate differences in symptom types. Importantly, the 3-year mean age gap is also lower than described in prior studies, which have reported an age difference between men and women up to 10 years at time of MI.^31–33^ The prior studies included patients from 1977 to 2010, implying that the proportion of women experiencing MI may have increased relatively in the last decades in comparison to men.

This study demonstrates the unmet need for improved patient identification and risk stratification in current clinical practice. Multiple techniques capturing risk on different levels on the pathway towards clinically overt ASCVD hold potential to improve identification of high-risk patients. Several studies have shown value of genetic risk markers, such as next-generation polygenic risk scores,^36^ but also novel plasma lipids and biomarkers such as lipoprotein(a) and plasma omics.^37,38^ Moving beyond risk stratification with risk factors and biomarkers to assessment of the actual phenotype of the atherosclerotic disease, imaging methods such as non-contrast computed tomography to measure coronary artery calcium and coronary computed tomography angiography hold major potential to improve screening for individuals at high risk for ASCVD.^39^ Nevertheless, these approaches have not been rigorously tested for application in clinical practice to date. Randomized clinical trials prospectively investigating the use of a screening-based approach to prevent ASCVD are needed to investigate the efficacy of such approaches in terms of event reductions and cost-effectiveness and such trials have recently commenced (NCT06112418).

### Limitations

This study had certain limitations. First, the patient population was limited to the United States, which potentially limits the generalizability to other countries. Second, data were collected from EHRs, medical claims, and pharmacy claims, which may have affected precision and could have resulted in sampling bias.^40^ Furthermore, patients might have been misclassified due to missing risk factor and medication data. International Classification of Diseases symptom classification could only be performed if patients visited their physician and documented symptoms and thus the reported symptom burden might be an underestimation. Nevertheless, the database covered 98% of health insurance plans, and the prevalence of risk factors as well as incidence of MI were in line with previous large observational studies and national reports.^20,22–26^ Third, given the size and nature of the database, individual lipid and blood pressure levels were unavailable for the majority of the cohort. Fourth, there were no data available for prior events before January 2014, so a small proportion of patients might still have experienced a prior cardiovascular event before 2014. However, if patients with prior MI had been included inadvertently, this would have increased the proportion of patients receiving preventive therapies and symptoms, thereby strengthening the current findings. Finally, the analysis was restricted to patients experiencing an in-hospital MI and did not include patients not presenting at the hospital, those with sudden cardiac death, or those with a silent MI.

## Conclusions

In a large real-world dataset, half of patients suffering first MI did not have any documented antecedent symptoms, and approximately 1 in 5 patients was without identified SMuRFs. There were lower rates of physician visits and use of preventive therapy among asymptomatic and younger patients. These findings highlight an urgent unmet need to improve identification and preventive treatment uptake in individuals at risk of MI.

## Supplementary Material

ehaf390_Supplementary_Data

## Declarations

### Data Availability

Individual participant data, after de-identification, that underlie the results reported in this article (text, tables, figures, and appendices) will not be shared publicly but are available to the reviewers and editors for verification of the reported results.

### Pre-registered Clinical Trial Number

Not supplied.

## References

[1] World Heart Federation . Deaths from cardiovascular disease surged 60% globally over the last 30 years: Report, Published 2023. https://world-heart-federation.org/news/deaths-from-cardiovascular-disease-surged-60-globally-over-the-last-30-years-report/ (13 July 2023, date last accessed).

[2] Arnett DK, Blumenthal RS, Albert MA, Buroker AB, Goldberger ZD, Hahn EJ, et al 2019 ACC/AHA guideline on the primary prevention of cardiovascular disease: a report of the American College of Cardiology/American Heart Association task force on clinical practice guidelines. Circulation 2019;140:e596–646. 10.1161/CIR.0000000000000678 30879355 PMC7734661

[3] Visseren FLJ, Mach F, Smulders YM, Carballo D, Koskinas KC, Bäck M, et al 2021 ESC guidelines on cardiovascular disease prevention in clinical practice. Eur Heart J 2021;42:3227–337. 10.1093/eurheartj/ehab484 34458905

[4] D’Agostino RB, Vasan RS, Pencina MJ, Wolf PA, Cobain M, Massaro JM, et al General cardiovascular risk profile for use in primary care: the framingham heart study. Circulation 2008;117:743–53. 10.1161/CIRCULATIONAHA.107.699579 18212285

[5] SCORE2 risk prediction algorithms: new models to estimate 10-year risk of cardiovascular disease in Europe. Eur Heart J 2021;42:2439–54. 10.1093/eurheartj/ehab309 34120177 PMC8248998

[6] Ray KK, Wright RS, Kallend D, Koenig W, Leiter LA, Raal FJ, et al Two phase 3 trials of inclisiran in patients with elevated LDL cholesterol. N Engl J Med 2020;382:1507–19. 10.1056/nejmoa1912387 32187462

[7] Bhatt DL, Steg PG, Miller M, Brinton EA, Jacobson TA, Ketchum SB, et al Cardiovascular risk reduction with icosapent ethyl for hypertriglyceridemia. N Engl J Med 2019;380:11–22. 10.1056/nejmoa1812792 30415628

[8] Packer M, Anker SD, Butler J, Filippatos G, Pocock SJ, Carson P, et al Cardiovascular and renal outcomes with empagliflozin in heart failure. N Engl J Med 2020;383:1413–24. 10.1056/nejmoa2022190 32865377

[9] Lincoff AM, Brown-Frandsen K, Colhoun HM, Deanfield J, Emerson SS, Esbjerg S, et al Semaglutide and cardiovascular outcomes in obesity without diabetes. N Engl J Med 2023;389:2221–32. 10.1056/nejmoa2307563 37952131

[10] Nissen SE, Lincoff AM, Brennan D, Ray KK, Mason D, Kastelein JJP, et al Bempedoic acid and cardiovascular outcomes in statin-intolerant patients. N Engl J Med 2023;388:1353–64. 10.1056/nejmoa2215024 36876740

[11] Eikelboom JW, Connolly SJ, Bosch J, Dagenais GR, Hart RG, Shestakovska O, et al Rivaroxaban with or without aspirin in stable cardiovascular disease. N Engl J Med 2017;377:1319–30. 10.1056/nejmoa1709118 28844192

[12] Harris L, L’Italien G, Kumar A, Seelam P, LaVallee C, Coric V, et al Real-world assessment of the relationship between migraine-related disability and healthcare costs in the United States. Headache 2022;62:473–81. 10.1111/head.14289 35343590 PMC9313575

[13] LaVallee C, Rascati KL, Gums TH. Antihypertensive agent utilization patterns among patients with uncontrolled hypertension in the United States. J Clin Hypertens 2020;22:2084–92. 10.1111/jch.14041 PMC8029865 32951318

[14] Zile MR, Desai AS, Agarwal R, Bharmi R, Dalal N, Adamson PB, et al Prognostic value of brain natriuretic peptide vs history of heart failure hospitalization in a large real-world population. Clin Cardiol 2020;43:1501–10. 10.1002/clc.23468 32949178 PMC7724209

[15] Davidson J, Banerjee A, Muzambi R, Smeeth L, Warren-Gash C. Validity of acute cardiovascular outcome diagnoses recorded in European electronic health records: a systematic review. Clin Epidemiol 2020;12:1095–111. 10.2147/CLEP.S265619 33116903 PMC7569174

[16] Saunders-Hastings P, Heong SW, Srichaikul J, Wong HL, Shoaibi A, Chada K, et al Acute myocardial infarction: development and application of an ICD-10-CM-based algorithm to a large U.S. Healthcare claims-based database. PLoS One 2021;16:e0253580. 10.1371/journal.pone.0253580 34197488 PMC8248590

[17] Tisdale RL, Fan J, Calma J, Cyr K, Podchiyska T, Stafford RS, et al Predictors of incident heart failure diagnosis setting: insights from the veterans affairs healthcare system. JACC Hear Fail 2023;11:347–58. 10.1016/j.jchf.2022.11.013 PMC10069381 36881392

[18] Sandhu AT, Tisdale RL, Rodriguez F, Stafford RS, Maron DJ, Hernandez-Boussard T, et al Disparity in the setting of incident heart failure diagnosis. Circ Hear Fail 2021;14:E008538. 10.1161/CIRCHEARTFAILURE.121.008538 PMC9070116 34311559

[19] Gulati M, Levy PD, Mukherjee D, Amsterdam E, Bhatt DL, Birtcher KK, et al 2021 AHA/ACC/ASE/CHEST/SAEM/SCCT/SCMR guideline for the evaluation and diagnosis of chest pain: a report of the American College of Cardiology/American Heart Association joint committee on clinical practice guidelines. J Am Coll Cardiol 2021;78:e187–285. 10.1016/j.jacc.2021.07.053 34756653

[20] Tsao CW, Aday AW, Almarzooq ZI, Anderson CAM, Arora P, Avery CL, et al Heart disease and stroke statistics—2023 update: a report from the American Heart Association. Circulation 2023;147:E93–E621. 10.1161/CIR.0000000000001123 36695182 PMC12135016

[21] Sandhu AT, Rodriguez F, Maron DJ, Heidenreich PA. Use of lipid-lowering therapy preceding first hospitalization for acute myocardial infarction or stroke. Am J Prev Cardiol 2022;12:100426. 10.1016/j.ajpc.2022.100426 36304918 PMC9593274

[22] Figtree GA, Vernon ST, Hadziosmanovic N, Sundström J, Alfredsson J, Arnott C, et al Mortality in STEMI patients without standard modifiable risk factors: a sex-disaggregated analysis of SWEDEHEART registry data. Lancet 2021;397:1085–94. 10.1016/S0140-6736(21)00272-5 33711294

[23] Figtree GA, Redfors B, Kozor R, Vernon ST, Grieve SM, Mazhar J, et al Clinical outcomes in patients with ST-segment elevation MI and No standard modifiable cardiovascular risk factors. JACC Cardiovasc Interv 2022;15:1167–75. 10.1016/j.jcin.2022.03.036 35680197

[24] Vernon ST, Coffey S, Bhindi R, Soo Hoo SY, Nelson GI, Ward MR, et al Increasing proportion of ST elevation myocardial infarction patients with coronary atherosclerosis poorly explained by standard modifiable risk factors. Eur J Prev Cardiol 2017;24:1824–30. 10.1177/2047487317720287 28703626

[25] Vernon ST, Coffey S, D'Souza M, Chow CK, Kilian J, Hyun K, et al ST-Segment–Elevation myocardial infarction (STEMI) patients without standard modifiable cardiovascular risk factors—how common are they, and what are their outcomes? J Am Heart Assoc 2019;8:e013296. 10.1161/JAHA.119.013296 31672080 PMC6898813

[26] Roth GA, Johnson CO, Abate KH, Abd-Allah F, Ahmed M, Alam K, et al The burden of cardiovascular diseases among US states, 1990-2016. JAMA Cardiol 2018;3:375–89. 10.1001/jamacardio.2018.0385 29641820 PMC6145754

[27] Figtree GA, Vernon ST, Harmer JA, Gray MP, Arnott C, Bachour E, et al Clinical pathway for coronary atherosclerosis in patients without conventional modifiable risk factors: JACC state-of-the-art review. J Am Coll Cardiol 2023;82:1343–59. 10.1016/j.jacc.2023.06.045 37730292 PMC10522922

[28] DeFilippis AP, Young R, Carrubba CJ, McEvoy JW, Budoff MJ, Blumenthal RS, et al An analysis of calibration and discrimination among multiple cardiovascular risk scores in a modern multiethnic cohort. Ann Intern Med 2015;162:266–75. 10.7326/M14-1281 25686167 PMC4414494

[29] Ridker PM, Buring JE, Rifai N, Cook NR. Development and validation of improved algorithms for the assessment of global cardiovascular risk in women: the reynolds risk score. J Am Med Assoc 2007;297:611–9. 10.1001/jama.297.6.611 17299196

[30] Cavallari I, Bhatt DL, Steg PG, Leiter LA, McGuire DK, Mosenzon O, et al Causes and risk factors for death in diabetes: a competing-risk analysis from the SAVOR-TIMI 53 trial. J Am Coll Cardiol 2021;77:1837–40. 10.1016/j.jacc.2021.02.030 33832610

[31] Millett ERC, Peters SAE, Woodward M. Sex differences in risk factors for myocardial infarction: cohort study of UK biobank participants. BMJ 2018;363:k4247. 10.1136/bmj.k4247 30404896 PMC6364292

[32] Schulte KJ, Mayrovitz HN. Myocardial infarction signs and symptoms: females vs. Males. Cureus 2023;15:e37522. 10.7759/cureus.37522 37193476 PMC10182740

[33] Albrektsen G, Heuch I, Løchen ML, Thelle DS, Wilsgaard T, Njølstad I, et al Lifelong gender gap in risk of incident myocardial infarction: the tromsø study. JAMA Intern Med 2016;176:1673–9. 10.1001/jamainternmed.2016.5451 27617629

[34] McSweeney JC, Cody M, O’Sullivan P, Elberson K, Moser DK, Garvin BJ. Women’s early warning symptoms of acute myocardial infarction. Circulation 2003;108:2619–23. 10.1161/01.CIR.0000097116.29625.7C 14597589

[35] Hofgren C, Karlson BW, Herlitz J. Prodromal symptoms in subsets of patients hospitalized for suspected acute myocardial infarction. Hear Lung—J Acute Crit Care 1995;24:3–10. 10.1016/S0147-9563(05)80089-5 7706097

[36] Khera AV, Chaffin M, Aragam KG, Haas ME, Roselli C, Choi SH, et al Genome-wide polygenic scores for common diseases identify individuals with risk equivalent to monogenic mutations. Nat Genet 2018;50:1219–24. 10.1038/s41588-018-0183-z 30104762 PMC6128408

[37] Kronenberg F, Mora S, Stroes ESG, Ference BA, Arsenault BJ, Berglund L, et al Lipoprotein(a) in atherosclerotic cardiovascular disease and aortic stenosis: a European atherosclerosis society consensus statement. Eur Heart J 2022;43:3925–46. 10.1093/eurheartj/ehac361 36036785 PMC9639807

[38] Nurmohamed NS, Kraaijenhof JM, Mayr M, Nicholls SJ, Koenig W, Catapano AL, et al Proteomics and lipidomics in atherosclerotic cardiovascular disease risk prediction. Eur Heart J 2023;44:1594–607. 10.1093/eurheartj/ehad161 36988179 PMC10163980

[39] Williams MC, Kwiecinski J, Doris M, McElhinney P, D’Souza MS, Cadet S, et al Low-attenuation noncalcified plaque on coronary computed tomography angiography predicts myocardial infarction: results from the multicenter SCOT-HEART trial (Scottish computed tomography of the HEART). Circulation 2020;141:1452–62. 10.1161/CIRCULATIONAHA.119.044720 32174130 PMC7195857

[40] Dahlen A, Charu V. Analysis of sampling bias in large health care claims databases. JAMA Netw Open 2023;6:e2249804. 10.1001/jamanetworkopen.2022.49804 36607640 PMC9857613

