# Supplementary material for: First myocardial infarction: risk factors, symptoms, and medical therapy
Source: Eur Heart J. 2025 Jul 3;46(38):3762–72. doi: 10.1093/eurheartj/ehaf390 (PMC12500329; doi:10.1093/eurheartj/ehaf390)
Supplement: ehaf390_Supplementary_Data [file ehaf390_supplementary_data.docx]

**ONLINE-ONLY SUPPLEMENT**

**First myocardial infarction: risk factors, symptoms, and medical therapy**

| **Content** | **Page** |
| --- | --- |
| Supplemental Figure 1. Flowchart of patient inclusion | 2 |
| Supplemental Table 1. ICD-10 and ICD-9 codes for myocardial infarction | 3 |
| Supplemental Table 2. ICD-10 codes for cardiovascular risk factors | 5 |
| Supplemental Table 3. ICD-10 codes for possible cardiac symptoms | 23 |
| Supplemental Table 4. Definition of preventive medical therapy | 24 |

**Supplemental Figure 1. Flowchart of patient inclusion**

**
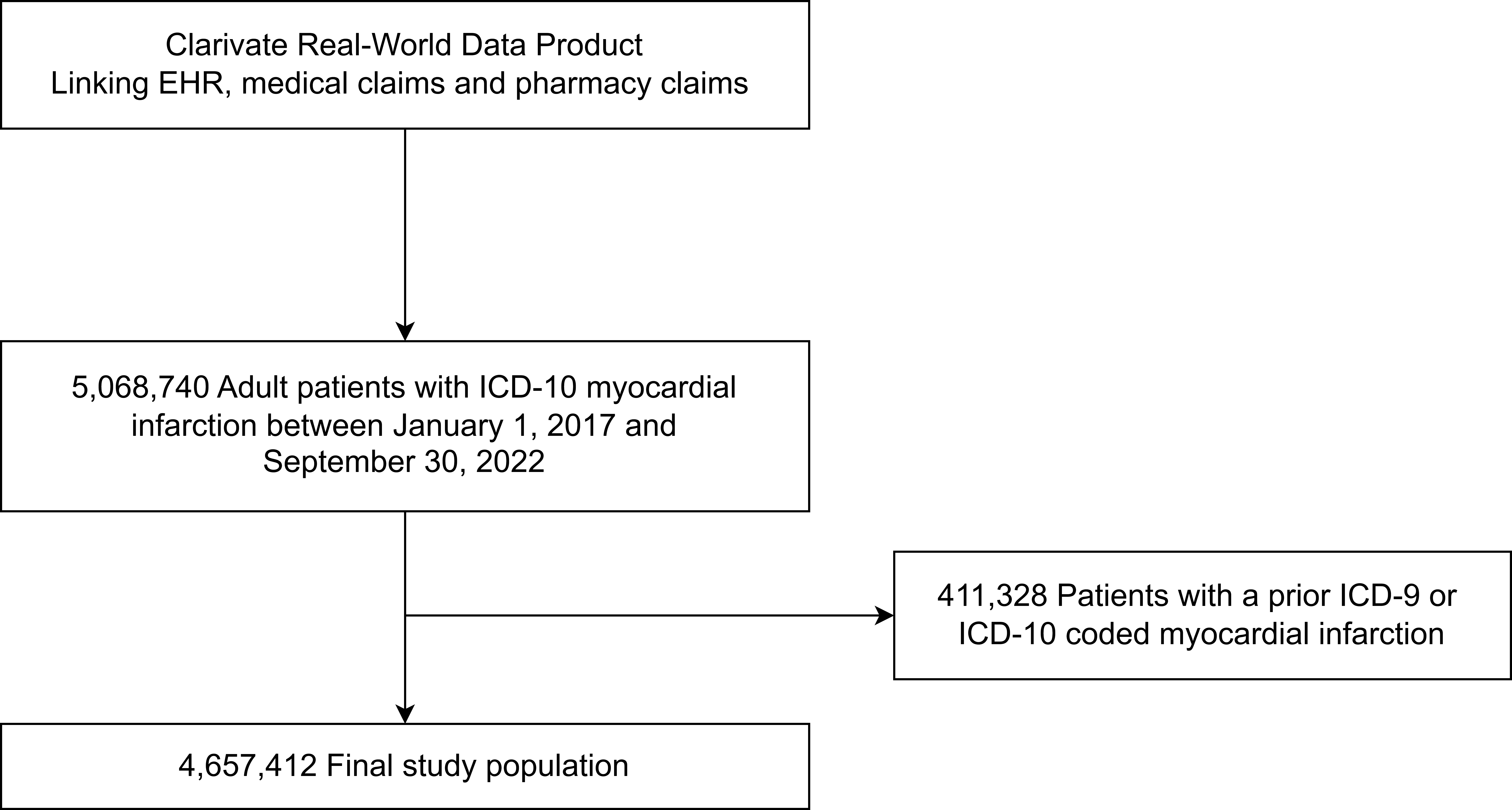
**

EHR, Electronic Health Records; ICD, International Classification of Diseases.

**Supplemental Table 1. ICD-10 and ICD-9 codes for myocardial infarction**

| **ICD** | **Code** | **Description** | **Groups** |
| --- | --- | --- | --- |
| ICD10 | I21.01 | ST Elevation (STEMI) Myocardial Infarction Involving Left Main Coronary Artery | STEMI |
| ICD10 | I21.02 | ST Elevation (STEMI) Myocardial Infarction Involving Left Anterior Descending Coronary Artery | STEMI |
| ICD10 | I21.09 | ST Elevation (STEMI) Myocardial Infarction Involving Other Coronary Artery Of Anterior Wall | STEMI |
| ICD10 | I21.11 | ST Elevation (STEMI) Myocardial Infarction Involving Right Coronary Artery | STEMI |
| ICD10 | I21.19 | ST Elevation (STEMI) Myocardial Infarction Involving Other Coronary Artery Of Inferior Wall | STEMI |
| ICD10 | I21.21 | ST Elevation (STEMI) Myocardial Infarction Involving Left Circumflex Coronary Artery | STEMI |
| ICD10 | I21.29 | ST Elevation (STEMI) Myocardial Infarction Involving Other Sites | STEMI |
| ICD10 | I21.3 | ST Elevation (STEMI) Myocardial Infarction Of Unspecified Site | STEMI |
| ICD10 | I21.4 | Non-ST Elevation (NSTEMI) Myocardial Infarction | NSTEMI |
| ICD9 | 41000 | Acute myocardial infarction of anterolateral wall, episode of care unspecified | - |
| ICD9 | 41001 | Acute myocardial infarction of anterolateral wall, initial episode of care | - |
| ICD9 | 41002 | Acute myocardial infarction of anterolateral wall, subsequent episode of care | - |
| ICD9 | 41010 | Acute myocardial infarction of other anterior wall, episode of care unspecified | - |
| ICD9 | 41011 | Acute myocardial infarction of other anterior wall, initial episode of care | - |
| ICD9 | 41011 | Acute myocardial infarction of other anterior wall, initial episode of care | - |
| ICD9 | 41011 | Acute myocardial infarction of other anterior wall, initial episode of care | - |
| ICD9 | 41012 | Acute myocardial infarction of anterolateral wall, subsequent episode of care | - |
| ICD9 | 41020 | Acute myocardial infarction of inferolateral wall, episode of care unspecified | - |
| ICD9 | 41021 | Acute myocardial infarction of inferolateral wall, initial episode of care | - |
| ICD9 | 41022 | Acute myocardial infarction of inferolateral wall, subsequent episode of care | - |
| ICD9 | 41030 | Acute myocardial infarction of inferoposterior wall, episode of care unspecified | - |
| ICD9 | 41031 | Acute myocardial infarction of inferoposterior wall, initial episode of care | - |
| ICD9 | 41032 | Acute myocardial infarction of inferoposterior wall, subsequent episode of care | - |
| ICD9 | 41040 | Acute myocardial infarction of other inferior wall, episode of care unspecified | - |
| ICD9 | 41041 | Acute myocardial infarction of other inferior wall, initial episode of care | - |
| ICD9 | 41042 | Acute myocardial infarction of other inferior wall, subsequent episode of care | - |
| ICD9 | 41050 | Acute myocardial infarction of other lateral wall, episode of care unspecified | - |
| ICD9 | 41051 | Acute myocardial infarction of other lateral wall, initial episode of care | - |
| ICD9 | 41052 | Acute myocardial infarction of other lateral wall, subsequent episode of care | - |
| ICD9 | 41060 | True posterior wall infarction, episode of care unspecified | - |
| ICD9 | 41061 | True posterior wall infarction, initial episode of care | - |
| ICD9 | 41062 | True posterior wall infarction, subsequent episode of care | - |
| ICD9 | 41070 | Subendocardial infarction, episode of care unspecified | - |
| ICD9 | 41071 | Subendocardial infarction, initial episode of care | - |
| ICD9 | 41072 | Subendocardial infarction, subsequent episode of care | - |
| ICD9 | 41080 | Acute myocardial infarction of other specified sites, episode of care unspecified | - |
| ICD9 | 41081 | Acute myocardial infarction of other specified sites, initial episode of care | - |
| ICD9 | 41081 | Acute myocardial infarction of other specified sites, initial episode of care | - |
| ICD9 | 41082 | Acute myocardial infarction of other specified sites, subsequent episode of care | - |
| ICD9 | 41090 | Acute myocardial infarction of unspecified site, episode of care unspecified | - |
| ICD9 | 41091 | Acute myocardial infarction of unspecified site, initial episode of care | - |
| ICD9 | 41092 | Acute myocardial infarction of unspecified site, subsequent episode of care | - |

ICD, International Classification of Diseases

**Supplemental Table 2. ICD-10 and ICD-9 codes for cardiovascular risk factors**

| **Code** | **Description** | **Risk factor** |
| --- | --- | --- |
| F10 | Alcohol related disorders | Alcohol abuse |
| F101 | Alcohol abuse | Alcohol abuse |
| F1010 | Alcohol abuse, uncomplicated | Alcohol abuse |
| F1011 | Alcohol abuse, in remission | Alcohol abuse |
| F1012 | Alcohol abuse with intoxication | Alcohol abuse |
| F10120 | Alcohol abuse with intoxication, uncomplicated | Alcohol abuse |
| F10121 | Alcohol abuse with intoxication delirium | Alcohol abuse |
| F10129 | Alcohol abuse with intoxication, unspecified | Alcohol abuse |
| F1014 | Alcohol abuse with alcohol-induced mood disorder | Alcohol abuse |
| F1015 | Alcohol abuse with alcohol-induced psychotic disorder | Alcohol abuse |
| F10150 | Alcohol abuse with alcohol-induced psychotic disorder with delusions | Alcohol abuse |
| F10151 | Alcohol abuse with alcohol-induced psychotic disorder with hallucinations | Alcohol abuse |
| F10159 | Alcohol abuse with alcohol-induced psychotic disorder, unspecified | Alcohol abuse |
| F1018 | Alcohol abuse with other alcohol-induced disorders | Alcohol abuse |
| F10180 | Alcohol abuse with alcohol-induced anxiety disorder | Alcohol abuse |
| F10181 | Alcohol abuse with alcohol-induced sexual dysfunction | Alcohol abuse |
| F10182 | Alcohol abuse with alcohol-induced sleep disorder | Alcohol abuse |
| F10188 | Alcohol abuse with other alcohol-induced disorder | Alcohol abuse |
| F1019 | Alcohol abuse with unspecified alcohol-induced disorder | Alcohol abuse |
| F102 | Alcohol dependence | Alcohol abuse |
| F1020 | Alcohol dependence, uncomplicated | Alcohol abuse |
| F1021 | Alcohol dependence, in remission | Alcohol abuse |
| F1022 | Alcohol dependence with intoxication | Alcohol abuse |
| F10220 | Alcohol dependence with intoxication, uncomplicated | Alcohol abuse |
| F10221 | Alcohol dependence with intoxication delirium | Alcohol abuse |
| F10229 | Alcohol dependence with intoxication, unspecified | Alcohol abuse |
| F1023 | Alcohol dependence with withdrawal | Alcohol abuse |
| F10230 | Alcohol dependence with withdrawal, uncomplicated | Alcohol abuse |
| F10231 | Alcohol dependence with withdrawal delirium | Alcohol abuse |
| F10232 | Alcohol dependence with withdrawal with perceptual disturbance | Alcohol abuse |
| F10239 | Alcohol dependence with withdrawal, unspecified | Alcohol abuse |
| F1024 | Alcohol dependence with alcohol-induced mood disorder | Alcohol abuse |
| F1025 | Alcohol dependence with alcohol-induced psychotic disorder | Alcohol abuse |
| F10250 | Alcohol dependence with alcohol-induced psychotic disorder with delusions | Alcohol abuse |
| F10251 | Alcohol dependence with alcohol-induced psychotic disorder with hallucinations | Alcohol abuse |
| F10259 | Alcohol dependence with alcohol-induced psychotic disorder, unspecified | Alcohol abuse |
| F1026 | Alcohol dependence with alcohol-induced persisting amnestic disorder | Alcohol abuse |
| F1027 | Alcohol dependence with alcohol-induced persisting dementia | Alcohol abuse |
| F1028 | Alcohol dependence with other alcohol-induced disorders | Alcohol abuse |
| F10280 | Alcohol dependence with alcohol-induced anxiety disorder | Alcohol abuse |
| F10281 | Alcohol dependence with alcohol-induced sexual dysfunction | Alcohol abuse |
| F10282 | Alcohol dependence with alcohol-induced sleep disorder | Alcohol abuse |
| F10288 | Alcohol dependence with other alcohol-induced disorder | Alcohol abuse |
| F1029 | Alcohol dependence with unspecified alcohol-induced disorder | Alcohol abuse |
| F1092 | Alcohol use, unspecified with intoxication | Alcohol abuse |
| F10920 | Alcohol use, unspecified with intoxication, uncomplicated | Alcohol abuse |
| F10921 | Alcohol use, unspecified with intoxication delirium | Alcohol abuse |
| F10929 | Alcohol use, unspecified with intoxication, unspecified | Alcohol abuse |
| F1094 | Alcohol use, unspecified with alcohol-induced mood disorder | Alcohol abuse |
| F1095 | Alcohol use, unspecified with alcohol-induced psychotic disorder | Alcohol abuse |
| F10950 | Alcohol use, unspecified with alcohol-induced psychotic disorder with delusions | Alcohol abuse |
| F10951 | Alcohol use, unspecified with alcohol-induced psychotic disorder with hallucinations | Alcohol abuse |
| F10959 | Alcohol use, unspecified with alcohol-induced psychotic disorder, unspecified | Alcohol abuse |
| F1096 | Alcohol use, unspecified with alcohol-induced persisting amnestic disorder | Alcohol abuse |
| F1097 | Alcohol use, unspecified with alcohol-induced persisting dementia | Alcohol abuse |
| F1098 | Alcohol use, unspecified with other alcohol-induced disorders | Alcohol abuse |
| F10980 | Alcohol use, unspecified with alcohol-induced anxiety disorder | Alcohol abuse |
| F10981 | Alcohol use, unspecified with alcohol-induced sexual dysfunction | Alcohol abuse |
| F10982 | Alcohol use, unspecified with alcohol-induced sleep disorder | Alcohol abuse |
| F10988 | Alcohol use, unspecified with other alcohol-induced disorder | Alcohol abuse |
| F1099 | Alcohol use, unspecified with unspecified alcohol-induced disorder | Alcohol abuse |
| Y90 | Evidence of alcohol involvement determined by blood alcohol level | Alcohol abuse |
| Z683 | Body mass index [BMI] 30-39, adult | Obesity |
| Z6830 | Body mass index [BMI] 30.0-30.9, adult | Obesity |
| Z6831 | Body mass index [BMI] 31.0-31.9, adult | Obesity |
| Z6832 | Body mass index [BMI] 32.0-32.9, adult | Obesity |
| Z6833 | Body mass index [BMI] 33.0-33.9, adult | Obesity |
| Z6834 | Body mass index [BMI] 34.0-34.9, adult | Obesity |
| Z6835 | Body mass index [BMI] 35.0-35.9, adult | Obesity |
| Z6836 | Body mass index [BMI] 36.0-36.9, adult | Obesity |
| Z6837 | Body mass index [BMI] 37.0-37.9, adult | Obesity |
| Z6838 | Body mass index [BMI] 38.0-38.9, adult | Obesity |
| Z6839 | Body mass index [BMI] 39.0-39.9, adult | Obesity |
| Z684 | Body mass index [BMI] 40 or greater, adult | Obesity |
| Z6841 | Body mass index [BMI] 40.0-44.9, adult | Obesity |
| Z6842 | Body mass index [BMI] 45.0-49.9, adult | Obesity |
| Z6843 | Body mass index [BMI] 50.0-59.9, adult | Obesity |
| Z6844 | Body mass index [BMI] 60.0-69.9, adult | Obesity |
| Z6845 | Body mass index [BMI] 70 or greater, adult | Obesity |
| E78 | Disorders of lipoprotein metabolism and other lipidemias | Dyslipidemia |
| E780 | Pure hypercholesterolemia | Dyslipidemia |
| E7800 | Unspecified | Dyslipidemia |
| E781 | Pure hypertriglyceridemia (Elevated fasting triglycerides) | Dyslipidemia |
| E782 | Mixed hyperlipidemia | Dyslipidemia |
| E783 | Hyperchylomicronemia | Dyslipidemia |
| E784 | Other hyperlipidemia | Dyslipidemia |
| E7841 | Elevated Lipoprotein(a) | Dyslipidemia |
| E7849 | Other hyperlipidemia | Dyslipidemia |
| E785 | Hyperlipidemia, unspecified | Dyslipidemia |
| E78.01 | Familial hypercholesterolemia | Dyslipidemia |
| Z8249 | Family history of ischemic heart disease and other diseases of the circulatory system | Family history of MI |
| Z824 | Family history of ischemic heart disease and other diseases of the circulatory system | Family history of MI |
| Z8241 | Family history of sudden cardiac death | Family history of MI |
| O240 | Pre-existing type 1 diabetes mellitus, in pregnancy, childbirth and the puerperium | Gestational diabetes |
| O2401 | Pre-existing type 1 diabetes mellitus, in pregnancy | Gestational diabetes |
| O24011 | Pre-existing type 1 diabetes mellitus, in pregnancy first trimester | Gestational diabetes |
| O24012 | Pre-existing type 1 diabetes mellitus, in pregnancy second trimester | Gestational diabetes |
| O24013 | Pre-existing type 1 diabetes mellitus, in pregnancy third trimester | Gestational diabetes |
| O24019 | Pre-existing type 1 diabetes mellitus, in pregnancy unspecified trimester | Gestational diabetes |
| O2402 | Pre-existing type 1 diabetes mellitus, in childbirth | Gestational diabetes |
| O2403 | Pre-existing type 1 diabetes mellitus, in the puerperium | Gestational diabetes |
| O241 | Pre-existing type 2 diabetes mellitus, in pregnancy, childbirth and the puerperium | Gestational diabetes |
| O2411 | Pre-existing type 2 diabetes mellitus, in pregnancy | Gestational diabetes |
| O24111 | Pre-existing type 2 diabetes mellitus, in pregnancy first trimester | Gestational diabetes |
| O24112 | Pre-existing type 2 diabetes mellitus, in pregnancy second trimester | Gestational diabetes |
| O24113 | Pre-existing type 2 diabetes mellitus, in pregnancy third trimester | Gestational diabetes |
| O24119 | Pre-existing type 2 diabetes mellitus, in pregnancy unspecified trimester | Gestational diabetes |
| O2412 | Pre-existing type 2 diabetes mellitus, in childbirth | Gestational diabetes |
| O2413 | Pre-existing type 2 diabetes mellitus, in the puerperium | Gestational diabetes |
| O243 | Unspecified pre-existing diabetes mellitus in pregnancy, childbirth and the puerperium | Gestational diabetes |
| O2431 | Unspecified pre-existing diabetes mellitus in pregnancy | Gestational diabetes |
| O24311 | Unspecified pre-existing diabetes mellitus in pregnancy first trimester | Gestational diabetes |
| O24312 | Unspecified pre-existing diabetes mellitus in pregnancy second trimester | Gestational diabetes |
| O24313 | Unspecified pre-existing diabetes mellitus in pregnancy third trimester | Gestational diabetes |
| O24319 | Unspecified pre-existing diabetes mellitus in pregnancy unspecified trimester | Gestational diabetes |
| O2432 | Unspecified pre-existing diabetes mellitus in childbirth | Gestational diabetes |
| O2433 | Unspecified pre-existing diabetes mellitus in the puerperium | Gestational diabetes |
| O244 | Gestational diabetes mellitus | Gestational diabetes |
| O2441 | Gestational diabetes mellitus in pregnancy | Gestational diabetes |
| O24410 | Gestational diabetes mellitus in pregnancy diet controlled | Gestational diabetes |
| O24414 | Gestational diabetes mellitus in pregnancy insulin controlled | Gestational diabetes |
| O24415 | Gestational diabetes mellitus in pregnancy controlled by oral hypoglycemic drugs | Gestational diabetes |
| O24419 | Gestational diabetes mellitus in pregnancy unspecified control | Gestational diabetes |
| O2442 | Gestational diabetes mellitus in childbirth | Gestational diabetes |
| O24420 | Gestational diabetes mellitus in childbirth diet controlled | Gestational diabetes |
| O24424 | Gestational diabetes mellitus in childbirth insulin controlled | Gestational diabetes |
| O24425 | Gestational diabetes mellitus in childbirth controlled by oral hypoglycemic drugs | Gestational diabetes |
| O24429 | Gestational diabetes mellitus in childbirth unspecified control | Gestational diabetes |
| O2443 | Gestational diabetes mellitus in the puerperium | Gestational diabetes |
| O24430 | Gestational diabetes mellitus in the puerperium diet controlled | Gestational diabetes |
| O24434 | Gestational diabetes mellitus in the puerperium insulin controlled | Gestational diabetes |
| O24435 | Gestational diabetes mellitus in puerperium, controlled by oral hypoglycemic drugs | Gestational diabetes |
| O24439 | Gestational diabetes mellitus in puerperium, controlled by oral hypoglycemic drugs unspecified control | Gestational diabetes |
| O248 | Other pre-existing diabetes mellitus in pregnancy, childbirth, and the puerperium | Gestational diabetes |
| O2481 | Other pre-existing diabetes mellitus in pregnancy | Gestational diabetes |
| O24811 | Other pre-existing diabetes mellitus in pregnancy first trimester | Gestational diabetes |
| O24812 | Other pre-existing diabetes mellitus in pregnancy second trimester | Gestational diabetes |
| O24813 | Other pre-existing diabetes mellitus in pregnancy third trimester | Gestational diabetes |
| O24819 | Other pre-existing diabetes mellitus in pregnancy unspecified trimester | Gestational diabetes |
| O2482 | Other pre-existing diabetes mellitus in childbirth | Gestational diabetes |
| O2483 | Other pre-existing diabetes mellitus in the puerperium | Gestational diabetes |
| O249 | Unspecified diabetes mellitus in pregnancy, childbirth and the puerperium | Gestational diabetes |
| O2491 | Unspecified diabetes mellitus in pregnancy | Gestational diabetes |
| O24911 | Unspecified diabetes mellitus in pregnancy first trimester | Gestational diabetes |
| O24912 | Unspecified diabetes mellitus in pregnancy second trimester | Gestational diabetes |
| O24913 | Unspecified diabetes mellitus in pregnancy third trimester | Gestational diabetes |
| O24919 | Unspecified diabetes mellitus in pregnancy unspecified trimester | Gestational diabetes |
| O2492 | Unspecified diabetes mellitus in childbirth | Gestational diabetes |
| O2493 | Unspecified diabetes mellitus in the puerperium | Gestational diabetes |
| I10 | Hypertension (arterial)(benign)(essential)(malignant)(primary)(systemic) | Hypertension |
| I1310 | Hypertensive heart and chronic kidney disease without heart failure with stage 1 through stage 4 chronic kidney disease, or unspecified chronic kidney disease, | Hypertension |
| I1311 | Hypertensive heart and chronic kidney disease without heart failure with stage 5 chronic kidney disease, or end-stage renal disease, | Hypertension |
| I132 | Hypertensive heart and chronic kidney disease with heart failure and with stage 5 chronic kidney disease, or end-stage renal disease | Hypertension |
| I11 | Hypertensive heart disease | Hypertension |
| I11.0 | Hypertensive heart disease with (congestive) heart failure | Hypertension |
| I11.9 | Hypertensive heart disease without (congestive) heart failure | Hypertension |
| I13.0 | Hypertensive heart and chronic kidney disease with heart failure and with stage 1 through 4 chronic kidney disease, or unspecified chronic kidney disease, | Hypertension |
| Z7722 | Contact with and (suspected) exposure to environmental tobacco smoke (acute) (chronic) | Nicotine Dependence |
| F17 | Nicotine dependence | Nicotine Dependence |
| F172 | Nicotine dependence | Nicotine Dependence |
| F1720 | Nicotine dependence, unspecified | Nicotine Dependence |
| F17200 | Nicotine dependence, unspecified, uncomplicated | Nicotine Dependence |
| F17201 | Nicotine dependence, unspecified, in remission | Nicotine Dependence |
| F17203 | Nicotine dependence unspecified, with withdrawal | Nicotine Dependence |
| F17208 | Nicotine dependence, unspecified, with other nicotine-induced disorders | Nicotine Dependence |
| F17209 | Nicotine dependence, unspecified, with unspecified nicotine-induced disorders | Nicotine Dependence |
| F1721 | Nicotine dependence, cigarettes | Nicotine Dependence |
| F17210 | Nicotine dependence, cigarettes, uncomplicated | Nicotine Dependence |
| F17211 | Nicotine dependence, cigarettes, in remission | Nicotine Dependence |
| F17213 | Nicotine dependence, cigarettes, with withdrawal | Nicotine Dependence |
| F17218 | Nicotine dependence, cigarettes, with other nicotine-induced disorders | Nicotine Dependence |
| F17219 | Nicotine dependence, cigarettes, with unspecified nicotine-induced disorders | Nicotine Dependence |
| F1722 | Nicotine dependence, chewing tobacco | Nicotine Dependence |
| F17220 | Nicotine dependence, chewing tobacco, uncomplicated | Nicotine Dependence |
| F17221 | Nicotine dependence, chewing tobacco, in remission | Nicotine Dependence |
| F17223 | Nicotine dependence, chewing tobacco, with withdrawal | Nicotine Dependence |
| F17228 | Nicotine dependence, chewing tobacco, with other nicotine-induced disorders | Nicotine Dependence |
| F17229 | Nicotine dependence, chewing tobacco, with unspecified nicotine-induced disorders | Nicotine Dependence |
| F1729 | Nicotine dependence, other tobacco product | Nicotine Dependence |
| F17290 | Nicotine dependence, other tobacco product, uncomplicated | Nicotine Dependence |
| F17291 | Nicotine dependence, other tobacco product, in remission | Nicotine Dependence |
| F17293 | Nicotine dependence, other tobacco product, with withdrawal | Nicotine Dependence |
| F17298 | Nicotine dependence, other tobacco product, with other nicotine-induced disorders | Nicotine Dependence |
| F17299 | Nicotine dependence, other tobacco product, with unspecified nicotine-induced disorders | Nicotine Dependence |
| Z720 | Tobacco use | Nicotine Dependence |
| Z87891 | Personal history of nicotine dependence | Nicotine Dependence |
| E66 | Overweight and obesity | Obesity |
| E660 | Obesity due to excess calories | Obesity |
| E6601 | Morbid (severe) obesity due to excess calories | Obesity |
| E6609 | Other obesity due to excess calories | Obesity |
| E661 | Drug-induced obesity | Obesity |
| E662 | Morbid (severe) obesity with alveolar hypoventilation | Obesity |
| E663 | Overweight | Obesity |
| E668 | Other obesity | Obesity |
| E669 | Obesity, unspecified | Obesity |
| E10 | Type 1 diabetes mellitus | Type 1 diabetes |
| E101 | Type 1 diabetes mellitus with ketoacidosis | Type 1 diabetes |
| E1010 | Type 1 diabetes mellitus with ketoacidosis without coma | Type 1 diabetes |
| E1011 | Type 1 diabetes mellitus with ketoacidosis with coma | Type 1 diabetes |
| E102 | Type 1 diabetes mellitus with kidney complications | Type 1 diabetes |
| E1021 | Type 1 diabetes mellitus with diabetic nephropathy | Type 1 diabetes |
| E1022 | Type 1 diabetes mellitus with diabetic chronic kidney disease | Type 1 diabetes |
| E1029 | Type 1 diabetes mellitus with other diabetic kidney complication | Type 1 diabetes |
| E103 | Type 1 diabetes mellitus with ophthalmic complications | Type 1 diabetes |
| E1031 | Type 1 diabetes mellitus with unspecified diabetic retinopathy | Type 1 diabetes |
| E10311 | Type 1 diabetes mellitus with unspecified diabetic retinopathy with macular edema | Type 1 diabetes |
| E10319 | Type 1 diabetes mellitus with unspecified diabetic retinopathy without macular edema | Type 1 diabetes |
| E1032 | Type 1 diabetes mellitus with mild nonproliferative diabetic retinopathy | Type 1 diabetes |
| E10321 | Type 1 diabetes mellitus with mild nonproliferative diabetic retinopathy with macular edema | Type 1 diabetes |
| E103211 | Type 1 diabetes mellitus with mild nonproliferative diabetic retinopathy with macular edema_ right eye | Type 1 diabetes |
| E103212 | Type 1 diabetes mellitus with mild nonproliferative diabetic retinopathy with macular edema_ left eye | Type 1 diabetes |
| E103213 | Type 1 diabetes mellitus with mild nonproliferative diabetic retinopathy with macular edema_ bilateral | Type 1 diabetes |
| E103219 | Type 1 diabetes mellitus with mild nonproliferative diabetic retinopathy with macular edema_ unspecified eye | Type 1 diabetes |
| E10329 | Type 1 diabetes mellitus with mild nonproliferative diabetic retinopathy without macular edema | Type 1 diabetes |
| E103291 | Type 1 diabetes mellitus with mild nonproliferative diabetic retinopathy without macular edema_ right eye | Type 1 diabetes |
| E103292 | Type 1 diabetes mellitus with mild nonproliferative diabetic retinopathy without macular edema_ left eye | Type 1 diabetes |
| E103293 | Type 1 diabetes mellitus with mild nonproliferative diabetic retinopathy without macular edema_ bilateral | Type 1 diabetes |
| E103299 | Type 1 diabetes mellitus with mild nonproliferative diabetic retinopathy without macular edema_ unspecified eye | Type 1 diabetes |
| E1033 | Type 1 diabetes mellitus with moderate nonproliferative diabetic retinopathy | Type 1 diabetes |
| E10331 | Type 1 diabetes mellitus with moderate nonproliferative diabetic retinopathy with macular edema | Type 1 diabetes |
| E103311 | Type 1 diabetes mellitus with moderate nonproliferative diabetic retinopathy with macular edema_ right eye | Type 1 diabetes |
| E103312 | Type 1 diabetes mellitus with moderate nonproliferative diabetic retinopathy with macular edema_ left eye | Type 1 diabetes |
| E103313 | Type 1 diabetes mellitus with moderate nonproliferative diabetic retinopathy with macular edema_ bilateral | Type 1 diabetes |
| E103319 | Type 1 diabetes mellitus with moderate nonproliferative diabetic retinopathy with macular edema_ unspecified eye | Type 1 diabetes |
| E10339 | Type 1 diabetes mellitus with moderate nonproliferative diabetic retinopathy without macular edema | Type 1 diabetes |
| E103391 | Type 1 diabetes mellitus with moderate nonproliferative diabetic retinopathy without macular edema_ right eye | Type 1 diabetes |
| E103392 | Type 1 diabetes mellitus with moderate nonproliferative diabetic retinopathy without macular edema_ left eye | Type 1 diabetes |
| E103393 | Type 1 diabetes mellitus with moderate nonproliferative diabetic retinopathy without macular edema_ bilateral | Type 1 diabetes |
| E103399 | Type 1 diabetes mellitus with moderate nonproliferative diabetic retinopathy without macular edema_ unspecified eye | Type 1 diabetes |
| E1034 | Type 1 diabetes mellitus with severe nonproliferative diabetic retinopathy | Type 1 diabetes |
| E10341 | Type 1 diabetes mellitus with severe nonproliferative diabetic retinopathy with macular edema | Type 1 diabetes |
| E103411 | Type 1 diabetes mellitus with severe nonproliferative diabetic retinopathy with macular edema_ right eye | Type 1 diabetes |
| E103412 | Type 1 diabetes mellitus with severe nonproliferative diabetic retinopathy with macular edema_ left eye | Type 1 diabetes |
| E103413 | Type 1 diabetes mellitus with severe nonproliferative diabetic retinopathy with macular edema_ bilateral | Type 1 diabetes |
| E103419 | Type 1 diabetes mellitus with severe nonproliferative diabetic retinopathy with macular edema_ unspecified eye | Type 1 diabetes |
| E10349 | Type 1 diabetes mellitus with severe nonproliferative diabetic retinopathy without macular edema | Type 1 diabetes |
| E103491 | Type 1 diabetes mellitus with severe nonproliferative diabetic retinopathy without macular edema_ right eye | Type 1 diabetes |
| E103492 | Type 1 diabetes mellitus with severe nonproliferative diabetic retinopathy without macular edema_ left eye | Type 1 diabetes |
| E103493 | Type 1 diabetes mellitus with severe nonproliferative diabetic retinopathy without macular edema_ bilateral | Type 1 diabetes |
| E103499 | Type 1 diabetes mellitus with severe nonproliferative diabetic retinopathy without macular edema_ unspecified eye | Type 1 diabetes |
| E1035 | Type 1 diabetes mellitus with proliferative diabetic retinopathy | Type 1 diabetes |
| E10351 | Type 1 diabetes mellitus with proliferative diabetic retinopathy with macular edema | Type 1 diabetes |
| E103511 | Type 1 diabetes mellitus with proliferative diabetic retinopathy with macular edema_ right eye | Type 1 diabetes |
| E103512 | Type 1 diabetes mellitus with proliferative diabetic retinopathy with macular edema_ left eye | Type 1 diabetes |
| E103513 | Type 1 diabetes mellitus with proliferative diabetic retinopathy with macular edema_bilateral | Type 1 diabetes |
| E103519 | Type 1 diabetes mellitus with proliferative diabetic retinopathy with macular edema_ unspecified eye | Type 1 diabetes |
| E10352 | Type 1 diabetes mellitus with proliferative diabetic retinopathy with traction retinal detachment involving the macula | Type 1 diabetes |
| E103521 | Type 1 diabetes mellitus with proliferative diabetic retinopathy with traction retinal detachment involving the macula_ right eye | Type 1 diabetes |
| E103522 | Type 1 diabetes mellitus with proliferative diabetic retinopathy with traction retinal detachment involving the macula_ left eye | Type 1 diabetes |
| E103523 | Type 1 diabetes mellitus with proliferative diabetic retinopathy with traction retinal detachment involving the macula_ bilateral | Type 1 diabetes |
| E103529 | Type 1 diabetes mellitus with proliferative diabetic retinopathy with traction retinal detachment involving the macula_ unspecified eye | Type 1 diabetes |
| E10353 | Type 1 diabetes mellitus with proliferative diabetic retinopathy with traction retinal detachment not involving the macula | Type 1 diabetes |
| E103531 | Type 1 diabetes mellitus with proliferative diabetic retinopathy with traction retinal detachment not involving the macula_ right eye | Type 1 diabetes |
| E103532 | Type 1 diabetes mellitus with proliferative diabetic retinopathy with traction retinal detachment not involving the macula_ left eye | Type 1 diabetes |
| E103533 | Type 1 diabetes mellitus with proliferative diabetic retinopathy with traction retinal detachment not involving the macula_ bilateral | Type 1 diabetes |
| E103539 | Type 1 diabetes mellitus with proliferative diabetic retinopathy with traction retinal detachment not involving the macula_ unspecified eye | Type 1 diabetes |
| E10354 | Type 1 diabetes mellitus with proliferative diabetic retinopathy with combined traction retinal detachment and rhegmatogenous retinal detachment | Type 1 diabetes |
| E103541 | Type 1 diabetes mellitus with proliferative diabetic retinopathy with combined traction retinal detachment and rhegmatogenous retinal detachment_ right eye | Type 1 diabetes |
| E103542 | Type 1 diabetes mellitus with proliferative diabetic retinopathy with combined traction retinal detachment and rhegmatogenous retinal detachment_ left eye | Type 1 diabetes |
| E103543 | Type 1 diabetes mellitus with proliferative diabetic retinopathy with combined traction retinal detachment and rhegmatogenous retinal detachment_ bilateral | Type 1 diabetes |
| E103549 | Type 1 diabetes mellitus with proliferative diabetic retinopathy with combined traction retinal detachment and rhegmatogenous retinal detachment_ unspecified eye | Type 1 diabetes |
| E10355 | Type 1 diabetes mellitus with stable proliferative diabetic retinopathy | Type 1 diabetes |
| E103551 | Type 1 diabetes mellitus with stable proliferative diabetic retinopathy_ right eye | Type 1 diabetes |
| E103552 | Type 1 diabetes mellitus with stable proliferative diabetic retinopathy_ left eye | Type 1 diabetes |
| E103553 | Type 1 diabetes mellitus with stable proliferative diabetic retinopathy_ bilateral | Type 1 diabetes |
| E103559 | Type 1 diabetes mellitus with stable proliferative diabetic retinopathy_ unspecified eye | Type 1 diabetes |
| E10359 | Type 1 diabetes mellitus with proliferative diabetic retinopathy without macular edema | Type 1 diabetes |
| E103591 | Type 1 diabetes mellitus with proliferative diabetic retinopathy without macular edema_right eye | Type 1 diabetes |
| E103592 | Type 1 diabetes mellitus with proliferative diabetic retinopathy without macular edema_left eye | Type 1 diabetes |
| E103593 | Type 1 diabetes mellitus with proliferative diabetic retinopathy without macular edema_bilateral | Type 1 diabetes |
| E103599 | Type 1 diabetes mellitus with proliferative diabetic retinopathy without macular edema_unspecified eye | Type 1 diabetes |
| E1036 | Type 1 diabetes mellitus with diabetic cataract | Type 1 diabetes |
| E1037 | Type 1 diabetes mellitus with diabetic macular edema_ resolved following treatment | Type 1 diabetes |
| E1037X1 | Type 1 diabetes mellitus with diabetic macular edema_ resolved following treatment_ right eye | Type 1 diabetes |
| E1037X2 | Type 1 diabetes mellitus with diabetic macular edema_ resolved following treatment_ left eye | Type 1 diabetes |
| E1037X3 | Type 1 diabetes mellitus with diabetic macular edema_ resolved following treatment_ bilateral | Type 1 diabetes |
| E1037X9 | Type 1 diabetes mellitus with diabetic macular edema_ resolved following treatment_ unspecified eye | Type 1 diabetes |
| E1039 | Type 1 diabetes mellitus with other diabetic ophthalmic complication | Type 1 diabetes |
| E104 | Type 1 diabetes mellitus with neurological complications | Type 1 diabetes |
| E1040 | Type 1 diabetes mellitus with diabetic neuropathy_ unspecified | Type 1 diabetes |
| E1041 | Type 1 diabetes mellitus with diabetic mononeuropathy | Type 1 diabetes |
| E1042 | Type 1 diabetes mellitus with diabetic polyneuropathy | Type 1 diabetes |
| E1043 | Type 1 diabetes mellitus with diabetic autonomic (poly)neuropathy | Type 1 diabetes |
| E1044 | Type 1 diabetes mellitus with diabetic amyotrophy | Type 1 diabetes |
| E1049 | Type 1 diabetes mellitus with other diabetic neurological complication | Type 1 diabetes |
| E105 | Type 1 diabetes mellitus with circulatory complications | Type 1 diabetes |
| E1051 | Type 1 diabetes mellitus with diabetic peripheral angiopathy without gangrene | Type 1 diabetes |
| E1052 | Type 1 diabetes mellitus with diabetic peripheral angiopathy with gangrene | Type 1 diabetes |
| E1059 | Type 1 diabetes mellitus with other circulatory complications | Type 1 diabetes |
| E106 | Type 1 diabetes mellitus with other specified complications | Type 1 diabetes |
| E1061 | Type 1 diabetes mellitus with diabetic arthropathy | Type 1 diabetes |
| E10610 | Type 1 diabetes mellitus with diabetic neuropathic arthropathy | Type 1 diabetes |
| E10618 | Type 1 diabetes mellitus with other diabetic arthropathy | Type 1 diabetes |
| E1062 | Type 1 diabetes mellitus with skin complications | Type 1 diabetes |
| E10620 | Type 1 diabetes mellitus with diabetic dermatitis | Type 1 diabetes |
| E10621 | Type 1 diabetes mellitus with foot ulcer | Type 1 diabetes |
| E10622 | Type 1 diabetes mellitus with other skin ulcer | Type 1 diabetes |
| E10628 | Type 1 diabetes mellitus with other skin complications | Type 1 diabetes |
| E1063 | Type 1 diabetes mellitus with oral complications | Type 1 diabetes |
| E10630 | Type 1 diabetes mellitus with periodontal disease | Type 1 diabetes |
| E10638 | Type 1 diabetes mellitus with other oral complications | Type 1 diabetes |
| E1064 | Type 1 diabetes mellitus with hypoglycemia | Type 1 diabetes |
| E10641 | Type 1 diabetes mellitus with hypoglycemia with coma | Type 1 diabetes |
| E10649 | Type 1 diabetes mellitus with hypoglycemia without coma | Type 1 diabetes |
| E1065 | Type 1 diabetes mellitus with hyperglycemia | Type 1 diabetes |
| E1069 | Type 1 diabetes mellitus with other specified complication | Type 1 diabetes |
| E108 | Type 1 diabetes mellitus with unspecified complications | Type 1 diabetes |
| E109 | Type 1 diabetes mellitus without complications | Type 1 diabetes |
| E11 | Type 2 diabetes mellitus | Type 2 diabetes |
| E110 | Type 2 diabetes mellitus with hyperosmolarity | Type 2 diabetes |
| E1100 | Type 2 diabetes mellitus with hyperosmolarity without nonketotic hyperglycemic-hyperosmolar coma (NKHHC) | Type 2 diabetes |
| E1101 | Type 2 diabetes mellitus with hyperosmolarity with coma | Type 2 diabetes |
| E111 | Type 2 diabetes mellitus with ketoacidosis | Type 2 diabetes |
| E1110 | Type 2 diabetes mellitus with ketoacidosis without coma | Type 2 diabetes |
| E1111 | Type 2 diabetes mellitus with ketoacidosis with coma | Type 2 diabetes |
| E112 | Type 2 diabetes mellitus with kidney complications | Type 2 diabetes |
| E1121 | Type 2 diabetes mellitus with diabetic nephropathy | Type 2 diabetes |
| E1122 | Type 2 diabetes mellitus with diabetic chronic kidney disease | Type 2 diabetes |
| E1129 | Type 2 diabetes mellitus with other diabetic kidney complication | Type 2 diabetes |
| E113 | Type 2 diabetes mellitus with ophthalmic complications | Type 2 diabetes |
| E1131 | Type 2 diabetes mellitus with unspecified diabetic retinopathy | Type 2 diabetes |
| E11311 | Type 2 diabetes mellitus with unspecified diabetic retinopathy with macular edema | Type 2 diabetes |
| E11319 | Type 2 diabetes mellitus with unspecified diabetic retinopathy without macular edema | Type 2 diabetes |
| E1132 | Type 2 diabetes mellitus with mild nonproliferative diabetic retinopathy | Type 2 diabetes |
| E11321 | Type 2 diabetes mellitus with mild nonproliferative diabetic retinopathy with macular edema | Type 2 diabetes |
| E113211 | Type 2 diabetes mellitus with mild nonproliferative diabetic retinopathy with macular edema_ right eye | Type 2 diabetes |
| E113212 | Type 2 diabetes mellitus with mild nonproliferative diabetic retinopathy with macular edema_ left eye | Type 2 diabetes |
| E113213 | Type 2 diabetes mellitus with mild nonproliferative diabetic retinopathy with macular edema_ bilateral | Type 2 diabetes |
| E113219 | Type 2 diabetes mellitus with mild nonproliferative diabetic retinopathy with macular edema_ unspecified eye | Type 2 diabetes |
| E11329 | Type 2 diabetes mellitus with mild nonproliferative diabetic retinopathy without macular edema | Type 2 diabetes |
| E113291 | Type 2 diabetes mellitus with mild nonproliferative diabetic retinopathy without macular edema_ right eye | Type 2 diabetes |
| E113292 | Type 2 diabetes mellitus with mild nonproliferative diabetic retinopathy without macular edema_ left eye | Type 2 diabetes |
| E113293 | Type 2 diabetes mellitus with mild nonproliferative diabetic retinopathy without macular edema_ bilateral | Type 2 diabetes |
| E113299 | Type 2 diabetes mellitus with mild nonproliferative diabetic retinopathy without macular edema_ unspecified eye | Type 2 diabetes |
| E1133 | Type 2 diabetes mellitus with moderate nonproliferative diabetic retinopathy | Type 2 diabetes |
| E11331 | Type 2 diabetes mellitus with moderate nonproliferative diabetic retinopathy with macular edema | Type 2 diabetes |
| E113311 | Type 2 diabetes mellitus with moderate nonproliferative diabetic retinopathy with macular edema_ right eye | Type 2 diabetes |
| E113312 | Type 2 diabetes mellitus with moderate nonproliferative diabetic retinopathy with macular edema_ left eye | Type 2 diabetes |
| E113313 | Type 2 diabetes mellitus with moderate nonproliferative diabetic retinopathy with macular edema_ bilateral | Type 2 diabetes |
| E113319 | Type 2 diabetes mellitus with moderate nonproliferative diabetic retinopathy with macular edema_ unspecified eye | Type 2 diabetes |
| E11339 | Type 2 diabetes mellitus with moderate nonproliferative diabetic retinopathy without macular edema | Type 2 diabetes |
| E113391 | Type 2 diabetes mellitus with moderate nonproliferative diabetic retinopathy without macular edema_ right eye | Type 2 diabetes |
| E113392 | Type 2 diabetes mellitus with moderate nonproliferative diabetic retinopathy without macular edema_ left eye | Type 2 diabetes |
| E113393 | Type 2 diabetes mellitus with moderate nonproliferative diabetic retinopathy without macular edema_ bilateral | Type 2 diabetes |
| E113399 | Type 2 diabetes mellitus with moderate nonproliferative diabetic retinopathy without macular edema_ unspecified eye | Type 2 diabetes |
| E1134 | Type 2 diabetes mellitus with severe nonproliferative diabetic retinopathy | Type 2 diabetes |
| E11341 | Type 2 diabetes mellitus with severe nonproliferative diabetic retinopathy with macular edema | Type 2 diabetes |
| E113411 | Type 2 diabetes mellitus with severe nonproliferative diabetic retinopathy with macular edema_ right eye | Type 2 diabetes |
| E113412 | Type 2 diabetes mellitus with severe nonproliferative diabetic retinopathy with macular edema_ left eye | Type 2 diabetes |
| E113413 | Type 2 diabetes mellitus with severe nonproliferative diabetic retinopathy with macular edema_ bilateral | Type 2 diabetes |
| E113419 | Type 2 diabetes mellitus with severe nonproliferative diabetic retinopathy with macular edema_ unspecified eye | Type 2 diabetes |
| E11349 | Type 2 diabetes mellitus with severe nonproliferative diabetic retinopathy without macular edema | Type 2 diabetes |
| E113491 | Type 2 diabetes mellitus with severe nonproliferative diabetic retinopathy without macular edema_ right eye | Type 2 diabetes |
| E113492 | Type 2 diabetes mellitus with severe nonproliferative diabetic retinopathy without macular edema_ left eye | Type 2 diabetes |
| E113493 | Type 2 diabetes mellitus with severe nonproliferative diabetic retinopathy without macular edema_ bilateral | Type 2 diabetes |
| E113499 | Type 2 diabetes mellitus with severe nonproliferative diabetic retinopathy without macular edema_ unspecified eye | Type 2 diabetes |
| E1135 | Type 2 diabetes mellitus with proliferative diabetic retinopathy | Type 2 diabetes |
| E11351 | Type 2 diabetes mellitus with proliferative diabetic retinopathy with macular edema | Type 2 diabetes |
| E113511 | Type 2 diabetes mellitus with proliferative diabetic retinopathy with macular edema_ right eye | Type 2 diabetes |
| E113512 | Type 2 diabetes mellitus with proliferative diabetic retinopathy with macular edema_ left eye | Type 2 diabetes |
| E113513 | Type 2 diabetes mellitus with proliferative diabetic retinopathy with macular edema_ bilateral | Type 2 diabetes |
| E113519 | Type 2 diabetes mellitus with proliferative diabetic retinopathy with macular edema_ unspecified eye | Type 2 diabetes |
| E11352 | Type 2 diabetes mellitus with proliferative diabetic retinopathy with traction retinal detachment involving the macula | Type 2 diabetes |
| E113521 | Type 2 diabetes mellitus with proliferative diabetic retinopathy with traction retinal detachment involving the macula_ right eye | Type 2 diabetes |
| E113522 | Type 2 diabetes mellitus with proliferative diabetic retinopathy with traction retinal detachment involving the macula_ left eye | Type 2 diabetes |
| E113523 | Type 2 diabetes mellitus with proliferative diabetic retinopathy with traction retinal detachment involving the macula_ bilateral | Type 2 diabetes |
| E113529 | Type 2 diabetes mellitus with proliferative diabetic retinopathy with traction retinal detachment involving the macula_ unspecified eye | Type 2 diabetes |
| E11353 | Type 2 diabetes mellitus with proliferative diabetic retinopathy with traction retinal detachment not involving the macula | Type 2 diabetes |
| E113531 | Type 2 diabetes mellitus with proliferative diabetic retinopathy with traction retinal detachment not involving the macula_ right eye | Type 2 diabetes |
| E113532 | Type 2 diabetes mellitus with proliferative diabetic retinopathy with traction retinal detachment not involving the macula_ left eye | Type 2 diabetes |
| E113533 | Type 2 diabetes mellitus with proliferative diabetic retinopathy with traction retinal detachment not involving the macula_ bilateral | Type 2 diabetes |
| E113539 | Type 2 diabetes mellitus with proliferative diabetic retinopathy with traction retinal detachment not involving the macula_ unspecified eye | Type 2 diabetes |
| E11354 | Type 2 diabetes mellitus with proliferative diabetic retinopathy with combined traction retinal detachment and rhegmatogenous retinal detachment | Type 2 diabetes |
| E113541 | Type 2 diabetes mellitus with proliferative diabetic retinopathy with combined traction retinal detachment and rhegmatogenous retinal detachment_ right eye | Type 2 diabetes |
| E113542 | Type 2 diabetes mellitus with proliferative diabetic retinopathy with combined traction retinal detachment and rhegmatogenous retinal detachment_ left eye | Type 2 diabetes |
| E113543 | Type 2 diabetes mellitus with proliferative diabetic retinopathy with combined traction retinal detachment and rhegmatogenous retinal detachment_ bilateral | Type 2 diabetes |
| E113549 | Type 2 diabetes mellitus with proliferative diabetic retinopathy with combined traction retinal detachment and rhegmatogenous retinal detachment_ unspecified eye | Type 2 diabetes |
| E11355 | Type 2 diabetes mellitus with stable proliferative diabetic retinopathy | Type 2 diabetes |
| E113551 | Type 2 diabetes mellitus with stable proliferative diabetic retinopathy_ right eye | Type 2 diabetes |
| E113552 | Type 2 diabetes mellitus with stable proliferative diabetic retinopathy_ left eye | Type 2 diabetes |
| E113553 | Type 2 diabetes mellitus with stable proliferative diabetic retinopathy_ bilateral | Type 2 diabetes |
| E113559 | Type 2 diabetes mellitus with stable proliferative diabetic retinopathy_ unspecified eye | Type 2 diabetes |
| E11359 | Type 2 diabetes mellitus with proliferative diabetic retinopathy without macular edema | Type 2 diabetes |
| E113591 | Type 2 diabetes mellitus with proliferative diabetic retinopathy without macular edema_ right eye | Type 2 diabetes |
| E113592 | Type 2 diabetes mellitus with proliferative diabetic retinopathy without macular edema_ left eye | Type 2 diabetes |
| E113593 | Type 2 diabetes mellitus with proliferative diabetic retinopathy without macular edema_ bilateral | Type 2 diabetes |
| E113599 | Type 2 diabetes mellitus with proliferative diabetic retinopathy without macular edema_ unspecified eye | Type 2 diabetes |
| E1136 | Type 2 diabetes mellitus with diabetic cataract | Type 2 diabetes |
| E1137 | Type 2 diabetes mellitus with diabetic macular edema_ resolved following treatment | Type 2 diabetes |
| E1137X1 | Type 2 diabetes mellitus with diabetic macular edema_ resolved following treatment_ right eye | Type 2 diabetes |
| E1137X2 | Type 2 diabetes mellitus with diabetic macular edema_ resolved following treatment_ left eye | Type 2 diabetes |
| E1137X3 | Type 2 diabetes mellitus with diabetic macular edema_ resolved following treatment_ bilateral | Type 2 diabetes |
| E1137X9 | Type 2 diabetes mellitus with diabetic macular edema_ resolved following treatment_ unspecified eye | Type 2 diabetes |
| E1139 | Type 2 diabetes mellitus with other diabetic ophthalmic complication | Type 2 diabetes |
| E114 | Type 2 diabetes mellitus with neurological complications | Type 2 diabetes |
| E1140 | Type 2 diabetes mellitus with diabetic neuropathy_ unspecified | Type 2 diabetes |
| E1141 | Type 2 diabetes mellitus with diabetic mononeuropathy | Type 2 diabetes |
| E1142 | Type 2 diabetes mellitus with diabetic polyneuropathy | Type 2 diabetes |
| E1143 | Type 2 diabetes mellitus with diabetic autonomic (poly)neuropathy | Type 2 diabetes |
| E1144 | Type 2 diabetes mellitus with diabetic amyotrophy | Type 2 diabetes |
| E1149 | Type 2 diabetes mellitus with other diabetic neurological complication | Type 2 diabetes |
| E115 | Type 2 diabetes mellitus with circulatory complications | Type 2 diabetes |
| E1151 | Type 2 diabetes mellitus with diabetic peripheral angiopathy without gangrene | Type 2 diabetes |
| E1152 | Type 2 diabetes mellitus with diabetic peripheral angiopathy with gangrene | Type 2 diabetes |
| E1159 | Type 2 diabetes mellitus with other circulatory complications | Type 2 diabetes |
| E116 | Type 2 diabetes mellitus with other specified complications | Type 2 diabetes |
| E1161 | Type 2 diabetes mellitus with diabetic arthropathy | Type 2 diabetes |
| E11610 | Type 2 diabetes mellitus with diabetic neuropathic arthropathy | Type 2 diabetes |
| E11618 | Type 2 diabetes mellitus with other diabetic arthropathy | Type 2 diabetes |
| E1162 | Type 2 diabetes mellitus with skin complications | Type 2 diabetes |
| E11620 | Type 2 diabetes mellitus with diabetic dermatitis | Type 2 diabetes |
| E11621 | Type 2 diabetes mellitus with foot ulcer | Type 2 diabetes |
| E11622 | Type 2 diabetes mellitus with other skin ulcer | Type 2 diabetes |
| E11628 | Type 2 diabetes mellitus with other skin complications | Type 2 diabetes |
| E1163 | Type 2 diabetes mellitus with oral complications | Type 2 diabetes |
| E11630 | Type 2 diabetes mellitus with periodontal disease | Type 2 diabetes |
| E11638 | Type 2 diabetes mellitus with other oral complications | Type 2 diabetes |
| E1164 | Type 2 diabetes mellitus with hypoglycemia | Type 2 diabetes |
| E11641 | Type 2 diabetes mellitus with hypoglycemia with coma | Type 2 diabetes |
| E11649 | Type 2 diabetes mellitus with hypoglycemia without coma | Type 2 diabetes |
| E1165 | Type 2 diabetes mellitus with hyperglycemia | Type 2 diabetes |
| E1169 | Type 2 diabetes mellitus with other specified complication | Type 2 diabetes |
| E118 | Type 2 diabetes mellitus with unspecified complications | Type 2 diabetes |
| E119 | Type 2 diabetes mellitus without complications | Type 2 diabetes |

ICD, International Classification of Diseases

**Supplemental Table 3. ICD-10 codes for possible cardiac symptoms**

| **Code** | **Description** | **Symptom group** |
| --- | --- | --- |
| R00 | Abnormalities of heart beat | Arrhythmia |
| R000 | Tachycardia, unspecified | Arrhythmia |
| R001 | Bradycardia, unspecified | Arrhythmia |
| R002 | Palpitations | Arrhythmia |
| R531 | Weakness | Fatigue / Weakness |
| R538 | Other malaise and fatigue | Fatigue / Weakness |
| R5383 | Other fatigue | Fatigue / Weakness |
| R12 | Heartburn | Heartburn |
| K30 | Functional dyspepsia | Indigestion |
| R6884 | Jaw pain | Jaw pain |
| R42 | Dizziness and giddiness | Lightheadedness or sudden dizziness |
| I209 | Angina pectoris, unspecified (Ischaemic chest pain) | Chest pain |
| R071 | Chest pain on breathing | Chest pain |
| R072 | Precordial pain | Chest pain |
| R073 | Other chest pain | Chest pain |
| R074 | Chest pain, unspecified | Chest pain |
| R078 | Other chest pain | Chest pain |
| R0789 | Other chest pain | Chest pain |
| R079 | Chest pain, unspecified | Chest pain |

ICD, International Classification of Diseases

**Supplemental Table 4. Definition of preventive medical therapy**

| **Drug class** | **Drug name** |
| --- | --- |
| ACE Inhibitors | Amlodipine-Benazepril |
| ACE Inhibitors | Amlodipine-Benazepril Hydrochloride |
| ACE Inhibitors | Benazepril |
| ACE Inhibitors | Benazepril-Hydrochlorothiazide |
| ACE Inhibitors | Captopril |
| ACE Inhibitors | Captopril-Hydrochlorothiazide |
| ACE Inhibitors | Enalapril-Diltiazem |
| ACE Inhibitors | Enalapril-Felodipine |
| ACE Inhibitors | Enalapril-Hydrochlorothiazide |
| ACE Inhibitors | Enalapril Maleate |
| ACE Inhibitors | Enalapril Maleate-Hydrochlorothiazide |
| ACE Inhibitors | Enalaprilat |
| ACE Inhibitors | Fosinopril |
| ACE Inhibitors | Fosinopril-Hydrochlorothiazide |
| ACE Inhibitors | Lisinopril |
| ACE Inhibitors | Lisinopril-Hydrochlorothiazide |
| ACE Inhibitors | Moexipril |
| ACE Inhibitors | Moexipril-Hydrochloride |
| ACE Inhibitors | Moexipril-Hydrochlorothiazide |
| ACE Inhibitors | Moexipril-Hydrochlorothiazide-Hydrochlorothiazide |
| ACE Inhibitors | Perindopril |
| ACE Inhibitors | Perindopril Erbumine |
| ACE Inhibitors | Quinapril |
| ACE Inhibitors | Quinapril-Hydrochlorothiazide |
| ACE Inhibitors | Ramipril |
| ACE Inhibitors | Trandolapril |
| ACE Inhibitors | Trandolapril-Verapamil |
| Angiotensin II Receptor Blockers | Aliskiren-Valsartan |
| Angiotensin II Receptor Blockers | Amlodipine-Hydrochlorothiazide-Valsartan |
| Angiotensin II Receptor Blockers | Amlodipine-Olmesartan |
| Angiotensin II Receptor Blockers | Amlodipine-Valsartan |
| Angiotensin II Receptor Blockers | Azilsartan Med-Chlorthalidone |
| Angiotensin II Receptor Blockers | Azilsartan Medoxomil |
| Angiotensin II Receptor Blockers | Candesartan |
| Angiotensin II Receptor Blockers | Candesartan-Hydrochlorothiazide |
| Angiotensin II Receptor Blockers | Eprosartan |
| Angiotensin II Receptor Blockers | Eprosartan-Hydrochlorothiazide |
| Angiotensin II Receptor Blockers | Hydrochlorothiazide - Irbesartan |
| Angiotensin II Receptor Blockers | Irbesartan |
| Angiotensin II Receptor Blockers | Irbesartan-Hydrochlorothiazide |
| Angiotensin II Receptor Blockers | Losartan |
| Angiotensin II Receptor Blockers | Losartan-Hydrochlorothiazide |
| Angiotensin II Receptor Blockers | Nebivolol-Valsartan |
| Angiotensin II Receptor Blockers | Olmesartan |
| Angiotensin II Receptor Blockers | Olmesartan-Amlodipin-Hcthiazid |
| Angiotensin II Receptor Blockers | Olmesartan-Hydrochlorothiazide |
| Angiotensin II Receptor Blockers | Olmesartan Medoxomil |
| Angiotensin II Receptor Blockers | Telmisartan |
| Angiotensin II Receptor Blockers | Telmisartan-Amlodipine |
| Angiotensin II Receptor Blockers | Telmisartan-Hydrochlorothiazide |
| Angiotensin II Receptor Blockers | Valsartan |
| Angiotensin II Receptor Blockers | Valsartan-Hydrochlorothiazide |
| Antihyperglycemics | Acarbose |
| Antihyperglycemics | Acetohexamide |
| Antihyperglycemics | Albiglutide |
| Antihyperglycemics | Alogliptin |
| Antihyperglycemics | Alogliptin-Metformin |
| Antihyperglycemics | Alogliptin-Pioglitazone |
| Antihyperglycemics | Bromocriptine |
| Antihyperglycemics | Canagliflozin |
| Antihyperglycemics | Canagliflozin-Metformin |
| Antihyperglycemics | Chlorpropamide |
| Antihyperglycemics | Dapagliflozin |
| Antihyperglycemics | Dapagliflozin-Metformin |
| Antihyperglycemics | Dapagliflozin-Saxagliptin |
| Antihyperglycemics | Dulaglutide |
| Antihyperglycemics | Empaglifloz-Linaglip-Metformin |
| Antihyperglycemics | Empagliflozin |
| Antihyperglycemics | Empagliflozin-Metformin |
| Antihyperglycemics | Empagliflozin - Linagliptin |
| Antihyperglycemics | Ertugliflozin |
| Antihyperglycemics | Ertugliflozin-Metformin |
| Antihyperglycemics | Ertugliflozin-Sitagliptin |
| Antihyperglycemics | Exenatide |
| Antihyperglycemics | Glimepiride |
| Antihyperglycemics | Glimepiride - Rosiglitazone |
| Antihyperglycemics | Glipizide |
| Antihyperglycemics | Glipizide-Metformin |
| Antihyperglycemics | Glyburide |
| Antihyperglycemics | Glyburide-Metformin |
| Antihyperglycemics | Insulin |
| Antihyperglycemics | Insulin Aspart |
| Antihyperglycemics | Insulin Aspart Protamine-Insulin Aspart |
| Antihyperglycemics | Insulin Aspart U-100 |
| Antihyperglycemics | Insulin Beef |
| Antihyperglycemics | Insulin Degludec |
| Antihyperglycemics | Insulin Degludec-Liraglutide |
| Antihyperglycemics | Insulin Degludec Injection |
| Antihyperglycemics | Insulin Detemir |
| Antihyperglycemics | Insulin Glargine |
| Antihyperglycemics | Insulin Glargine-Lixisenatide |
| Antihyperglycemics | Insulin Glargine U-300 Conc |
| Antihyperglycemics | Insulin Glulisine |
| Antihyperglycemics | Insulin Human |
| Antihyperglycemics | Insulin Inhalation |
| Antihyperglycemics | Insulin Isophane |
| Antihyperglycemics | Insulin Isophane-Insulin Regular |
| Antihyperglycemics | Insulin Isophane - Insulin Regular |
| Antihyperglycemics | Insulin Isophane Beef |
| Antihyperglycemics | Insulin Isophane Pork Pure |
| Antihyperglycemics | Insulin Lispro |
| Antihyperglycemics | Insulin Lispro Protamin-Lispro |
| Antihyperglycemics | Insulin Nph-Regular Hum S-Syn |
| Antihyperglycemics | Insulin Nph-Regular Human |
| Antihyperglycemics | Insulin Nph Human Semi-Syn |
| Antihyperglycemics | Insulin Protamine Zn Beef |
| Antihyperglycemics | Insulin Protamine Zn Beef-Pork |
| Antihyperglycemics | Insulin Protamine Zn Beef Pure |
| Antihyperglycemics | Insulin Pump |
| Antihyperglycemics | Insulin Reg (Human) Buffered |
| Antihyperglycemics | Insulin Regular |
| Antihyperglycemics | Insulin Regular Human |
| Antihyperglycemics | Insulin Regular Pork |
| Antihyperglycemics | Insulin Zinc |
| Antihyperglycemics | Insulin Zinc Beef |
| Antihyperglycemics | Insulin Zinc Beef Purified |
| Antihyperglycemics | Insulin Zinc Ext Beef-Pork |
| Antihyperglycemics | Insulin Zinc Ext Beef Pure |
| Antihyperglycemics | Insulin Zinc Extended |
| Antihyperglycemics | Insulin Zinc Pork Purified |
| Antihyperglycemics | Insulin Zinc Prompt Beef |
| Antihyperglycemics | Insulin Zinc Prompt Beef-Pork |
| Antihyperglycemics | Insulin Zinc Prompt Pork Pure |
| Antihyperglycemics | Isophane Insulin |
| Antihyperglycemics | Linagliptin |
| Antihyperglycemics | Linagliptin-Metformin |
| Antihyperglycemics | Liraglutide |
| Antihyperglycemics | Lixisenatide |
| Antihyperglycemics | Metformin |
| Antihyperglycemics | Metformin-Saxagliptin |
| Antihyperglycemics | Metformin - Rosiglitazone |
| Antihyperglycemics | Metformin Er |
| Antihyperglycemics | Metformin Hydrochloride |
| Antihyperglycemics | Mifepristone |
| Antihyperglycemics | Miglitol |
| Antihyperglycemics | Nateglinide |
| Antihyperglycemics | Niacinamide |
| Antihyperglycemics | Novolin N Innolet |
| Antihyperglycemics | Nph, Human Insulin Isophane |
| Antihyperglycemics | Nsulin Isophane Beef Pure |
| Antihyperglycemics | Pioglitazone |
| Antihyperglycemics | Pioglitazone-Glimepiride |
| Antihyperglycemics | Pioglitazone-Metformin |
| Antihyperglycemics | Pramlintide |
| Antihyperglycemics | Repaglinide |
| Antihyperglycemics | Repaglinide-Metformin |
| Antihyperglycemics | Repaglinide-Metformin Hcl Tablets |
| Antihyperglycemics | Rosiglitazone |
| Antihyperglycemics | Saxagliptin |
| Antihyperglycemics | Semaglutide |
| Antihyperglycemics | Simvastatin-Sitagliptin |
| Antihyperglycemics | Sitagliptin |
| Antihyperglycemics | Sitagliptin-Metformin |
| Antihyperglycemics | Timolol Maleate-Hydrochlorothiazide |
| Antihyperglycemics | Tolazamide |
| Antihyperglycemics | Tolbutamide |
| Antihyperglycemics | Troglitazone |
| Antiplatelet Drugs | Abciximab |
| Antiplatelet Drugs | Acetylsalicylic Acid |
| Antiplatelet Drugs | Anagrelide |
| Antiplatelet Drugs | Aspirin |
| Antiplatelet Drugs | Aspirin-Dipyridamole |
| Antiplatelet Drugs | Aspirin-Omeprazole |
| Antiplatelet Drugs | Cilostazol |
| Antiplatelet Drugs | Clopidogrel |
| Antiplatelet Drugs | Dipyridamole |
| Antiplatelet Drugs | Eptifibatide |
| Antiplatelet Drugs | Kengreal |
| Antiplatelet Drugs | Prasugrel |
| Antiplatelet Drugs | Ticagrelor |
| Antiplatelet Drugs | Ticlopidine |
| Antiplatelet Drugs | Tirofiban |
| Antiplatelet Drugs | Vorapaxar |
| Beta Blockers | Acebutolol |
| Beta Blockers | Atenolol |
| Beta Blockers | Atenolol-Chlorthalidone |
| Beta Blockers | Atenolol Scopolamine |
| Beta Blockers | Betaxolol |
| Beta Blockers | Bisoprolol-Hydrochlorothiazide |
| Beta Blockers | Bisoprolol Fumarate |
| Beta Blockers | Brimonidine-Timolol |
| Beta Blockers | Carteolol |
| Beta Blockers | Carvedilol |
| Beta Blockers | Carvedilol Phosphate |
| Beta Blockers | Dorzolamide-Timolol |
| Beta Blockers | Esmolol |
| Beta Blockers | Insulin Detemir |
| Beta Blockers | Labetalol |
| Beta Blockers | Metoprolol |
| Beta Blockers | Metoprolol-Hydrochlorothiaz |
| Beta Blockers | Metoprolol-Hydrochlorothiazide |
| Beta Blockers | Metoprolol Er-Hydrochlorothiaz |
| Beta Blockers | Metoprolol Su-Hydrochlorothiaz |
| Beta Blockers | Metoprolol Succinate |
| Beta Blockers | Metoprolol Succinate Hydrochlorothiaz |
| Beta Blockers | Metoprolol Tartrate |
| Beta Blockers | Metoprolol Tartrate-Hydochlorothiazide |
| Beta Blockers | Nadolol |
| Beta Blockers | Nadolol-Bendroflumethiazide |
| Beta Blockers | Nebivolol |
| Beta Blockers | Normodyne-Trandate |
| Beta Blockers | Normodyne-Trandate-Dextrose |
| Beta Blockers | Penbutolol |
| Beta Blockers | Pindolol |
| Beta Blockers | Propranolol |
| Beta Blockers | Propranolol-Hydrochlorothiazid |
| Beta Blockers | Propranolol - Scopolamine |
| Beta Blockers | Sotalol |
| Beta Blockers | Timolol |
| Beta Blockers | Timolol-Brimonidi-Dorzolam(Pf) |
| Beta Blockers | Timolol Maleate |
| Beta Blockers | Timolol Maleate-Hydrochlorothiazide |
| Calcium Channel Blockers | Aliskiren-Amlodipine |
| Calcium Channel Blockers | Aliskiren-Amlodipine-Hydrochlorothiazide |
| Calcium Channel Blockers | Amlodipine |
| Calcium Channel Blockers | Amlodipine-Atorvastatin |
| Calcium Channel Blockers | Amlodipine-Celecoxib |
| Calcium Channel Blockers | Clevidipine |
| Calcium Channel Blockers | Diltiazem |
| Calcium Channel Blockers | Diltiazem Hcl |
| Calcium Channel Blockers | Diltiazem Malate |
| Calcium Channel Blockers | Felodipine |
| Calcium Channel Blockers | Isradipine |
| Calcium Channel Blockers | Levamlodipine |
| Calcium Channel Blockers | Meningococcal Vac A,C,Y,W-135 |
| Calcium Channel Blockers | Nicardipine |
| Calcium Channel Blockers | Nifedipine |
| Calcium Channel Blockers | Nimodipine |
| Calcium Channel Blockers | Nisoldipine |
| Calcium Channel Blockers | Typhoid Vaccine (Inactivated) |
| Calcium Channel Blockers | Verapamil |
| Diuretics | Acetazolamide |
| Diuretics | Acetazolamide Sodium |
| Diuretics | Amiloride |
| Diuretics | Amiloride-Hydrochlorothiazide |
| Diuretics | Ammonium Chloride |
| Diuretics | Bendroflumethiazide |
| Diuretics | Benzphetamine |
| Diuretics | Buchu-Cornsilk-Ch Grass-Hydran |
| Diuretics | Bumetanide |
| Diuretics | Chlorothiazide |
| Diuretics | Chlorothiazide Sodium |
| Diuretics | Chlorthalidone |
| Diuretics | Conivaptan |
| Diuretics | Conivaptan In 5 % Dextrose |
| Diuretics | Dichlorphenamide |
| Diuretics | Diuretics |
| Diuretics | Eplerenone |
| Diuretics | Ethacrynate Sodium |
| Diuretics | Ethacrynic Acid |
| Diuretics | Finerenone |
| Diuretics | Furosemide |
| Diuretics | Glycerin |
| Diuretics | Hydrochlorothiazide |
| Diuretics | Hydrochlorothiazide-Spironolactone |
| Diuretics | Hydroflumethiazide |
| Diuretics | Indapamide |
| Diuretics | Isosorbide |
| Diuretics | Mannitol |
| Diuretics | Methazolamide |
| Diuretics | Methyclothiazide |
| Diuretics | Metolazone |
| Diuretics | Pamabrom |
| Diuretics | Polythiazide |
| Diuretics | Spironolacton-Hydrochlorothiazide |
| Diuretics | Spironolactone |
| Diuretics | Tolvaptan |
| Diuretics | Torsemide |
| Diuretics | Triamterene |
| Diuretics | Triamterene-Hydrochlorothiazid |
| Diuretics | Trichlormethiazide |
| Diuretics | Unclassified Drugs |
| Diuretics | Urea |
| Diuretics | Urea (Diuretic) |
| Ezetimibe | Ezetimibe |
| Nitrate Vasodilator | Isosorbide Dinitrate |
| Nitrate Vasodilator | Isosorbide Mononitrate |
| Nitrate Vasodilator | Nitroglycerin |
| Oral Anticoagulants | Anisindione |
| Oral Anticoagulants | Apixaban |
| Oral Anticoagulants | Betrixaban |
| Oral Anticoagulants | Dabigatran |
| Oral Anticoagulants | Dicumarol |
| Oral Anticoagulants | Edoxaban |
| Oral Anticoagulants | Rivaroxaban |
| Oral Anticoagulants | Warfarin |
| Other Lipid Lowering Drugs | Cholestyramine |
| Other Lipid Lowering Drugs | Cholestyramine-Aspartame |
| Other Lipid Lowering Drugs | Niacin |
| PCSK9 inhibitors | Alirocumab |
| PCSK9 inhibitors | Evolocumab |
| Statins | Amlodipine-Atorvastatin |
| Statins | Aspirin-Pravastatin |
| Statins | Atorvastatin |
| Statins | Atorvastatin-Ezetimibe |
| Statins | Ezetimibe |
| Statins | Ezetimibe-Rosuvastatin |
| Statins | Ezetimibe-Simvastatin |
| Statins | Fluvastatin |
| Statins | Lovastatin |
| Statins | Niacin-Simvastatin |
| Statins | Niacin - Lovastatin |
| Statins | Pitavastatin |
| Statins | Pitavastatin Magnesium |
| Statins | Pravastatin |
| Statins | Rosuvastatin |
| Statins | Simvastatin |
| Statins | Simvastatin-Sitagliptin |
| Vasodilators | Acetaminophen |
| Vasodilators | Alprostadil |
| Vasodilators | Alprostadil In Sodium Chloride |
| Vasodilators | Amyl Nitrite |
| Vasodilators | Ergoloid |
| Vasodilators | Ergoloid Mesylates |
| Vasodilators | Ethaverine |
| Vasodilators | Human Immunoglobulin G |
| Vasodilators | Hydralazine |
| Vasodilators | Hydralazine-Hydrochlorothiazide |
| Vasodilators | Hydralazine-Hydrochlorothiazide-Reserpine |
| Vasodilators | Hydrochlorothiazide |
| Vasodilators | Hydrochlorothiazide-Reserpine |
| Vasodilators | Isosorbide-Hydralazine |
| Vasodilators | Isoxsuprine |
| Vasodilators | Minoxidil |
| Vasodilators | Nesiritide |
| Vasodilators | Papaverine |
| Vasodilators | Pavadyl Hydrochloride |
| Vasodilators | Phentolam-Alprostadil In Water |
| Vasodilators | Riociguat |
| Vasodilators | Sodium Nitroprusside |
| Vasodilators | Tacrolimus |
| Vasodilators | Triamterene-Hydrochlorothiazid |
| Vasodilators | Vericiguat |
| ACE Inhibitors | Amlodipine-Benazepril |
| ACE Inhibitors | Amlodipine-Benazepril Hydrochloride |
| ACE Inhibitors | Benazepril |
| ACE Inhibitors | Benazepril-Hydrochlorothiazide |
| ACE Inhibitors | Captopril |
| ACE Inhibitors | Captopril-Hydrochlorothiazide |
| ACE Inhibitors | Enalapril-Diltiazem |
| ACE Inhibitors | Enalapril-Felodipine |
| ACE Inhibitors | Enalapril-Hydrochlorothiazide |
| ACE Inhibitors | Enalapril Maleate |
| ACE Inhibitors | Enalapril Maleate-Hydrochlorothiazide |
| ACE Inhibitors | Enalaprilat |
| ACE Inhibitors | Fosinopril |
| ACE Inhibitors | Fosinopril-Hydrochlorothiazide |
| ACE Inhibitors | Lisinopril |
| ACE Inhibitors | Lisinopril-Hydrochlorothiazide |
| ACE Inhibitors | Moexipril |
| ACE Inhibitors | Moexipril-Hydrochloride |
| ACE Inhibitors | Moexipril-Hydrochlorothiazide |
| ACE Inhibitors | Moexipril-Hydrochlorothiazide-Hydrochlorothiazide |
| ACE Inhibitors | Perindopril |
| ACE Inhibitors | Perindopril Erbumine |
| ACE Inhibitors | Quinapril |
| ACE Inhibitors | Quinapril-Hydrochlorothiazide |
| ACE Inhibitors | Ramipril |
| ACE Inhibitors | Trandolapril |
| ACE Inhibitors | Trandolapril-Verapamil |
| Angiotensin II Receptor Blockers | Aliskiren-Valsartan |
| Angiotensin II Receptor Blockers | Amlodipine-Hydrochlorothiazide-Valsartan |
| Angiotensin II Receptor Blockers | Amlodipine-Olmesartan |
| Angiotensin II Receptor Blockers | Amlodipine-Valsartan |
| Angiotensin II Receptor Blockers | Azilsartan Med-Chlorthalidone |
| Angiotensin II Receptor Blockers | Azilsartan Medoxomil |
| Angiotensin II Receptor Blockers | Candesartan |
| Angiotensin II Receptor Blockers | Candesartan-Hydrochlorothiazide |
| Angiotensin II Receptor Blockers | Eprosartan |
| Angiotensin II Receptor Blockers | Eprosartan-Hydrochlorothiazide |
| Angiotensin II Receptor Blockers | Hydrochlorothiazide - Irbesartan |
| Angiotensin II Receptor Blockers | Irbesartan |
| Angiotensin II Receptor Blockers | Irbesartan-Hydrochlorothiazide |
| Angiotensin II Receptor Blockers | Losartan |
| Angiotensin II Receptor Blockers | Losartan-Hydrochlorothiazide |
| Angiotensin II Receptor Blockers | Nebivolol-Valsartan |
| Angiotensin II Receptor Blockers | Olmesartan |
| Angiotensin II Receptor Blockers | Olmesartan-Amlodipin-Hcthiazid |
| Angiotensin II Receptor Blockers | Olmesartan-Hydrochlorothiazide |
| Angiotensin II Receptor Blockers | Olmesartan Medoxomil |
| Angiotensin II Receptor Blockers | Telmisartan |
| Angiotensin II Receptor Blockers | Telmisartan-Amlodipine |
| Angiotensin II Receptor Blockers | Telmisartan-Hydrochlorothiazide |
| Angiotensin II Receptor Blockers | Valsartan |
| Angiotensin II Receptor Blockers | Valsartan-Hydrochlorothiazide |
| Antihyperglycemics | Acarbose |
| Antihyperglycemics | Acetohexamide |
| Antihyperglycemics | Albiglutide |
| Antihyperglycemics | Alogliptin |
| Antihyperglycemics | Alogliptin-Metformin |
| Antihyperglycemics | Alogliptin-Pioglitazone |
| Antihyperglycemics | Bromocriptine |
| Antihyperglycemics | Canagliflozin |
| Antihyperglycemics | Canagliflozin-Metformin |
| Antihyperglycemics | Chlorpropamide |
| Antihyperglycemics | Dapagliflozin |
| Antihyperglycemics | Dapagliflozin-Metformin |
| Antihyperglycemics | Dapagliflozin-Saxagliptin |
| Antihyperglycemics | Dulaglutide |
| Antihyperglycemics | Empaglifloz-Linaglip-Metformin |
| Antihyperglycemics | Empagliflozin |
| Antihyperglycemics | Empagliflozin-Metformin |
| Antihyperglycemics | Empagliflozin - Linagliptin |
| Antihyperglycemics | Ertugliflozin |
| Antihyperglycemics | Ertugliflozin-Metformin |
| Antihyperglycemics | Ertugliflozin-Sitagliptin |
| Antihyperglycemics | Exenatide |
| Antihyperglycemics | Glimepiride |
| Antihyperglycemics | Glimepiride - Rosiglitazone |
| Antihyperglycemics | Glipizide |
| Antihyperglycemics | Glipizide-Metformin |
| Antihyperglycemics | Glyburide |
| Antihyperglycemics | Glyburide-Metformin |
| Antihyperglycemics | Insulin |
| Antihyperglycemics | Insulin Aspart |
| Antihyperglycemics | Insulin Aspart Protamine-Insulin Aspart |
| Antihyperglycemics | Insulin Aspart U-100 |
| Antihyperglycemics | Insulin Beef |
| Antihyperglycemics | Insulin Degludec |
| Antihyperglycemics | Insulin Degludec-Liraglutide |
| Antihyperglycemics | Insulin Degludec Injection |
| Antihyperglycemics | Insulin Detemir |
| Antihyperglycemics | Insulin Glargine |
| Antihyperglycemics | Insulin Glargine-Lixisenatide |
